# Diabetes mellitus and tuberculosis, a systematic review and meta-analysis with sensitivity analysis for studies comparable for confounders

**DOI:** 10.1371/journal.pone.0261246

**Published:** 2021-12-10

**Authors:** Joseph Rodrigue Foe-Essomba, Sebastien Kenmoe, Serges Tchatchouang, Jean Thierry Ebogo-Belobo, Donatien Serge Mbaga, Cyprien Kengne-Ndé, Gadji Mahamat, Ginette Irma Kame-Ngasse, Efietngab Atembeh Noura, Chris Andre Mbongue Mikangue, Alfloditte Flore Feudjio, Jean Bosco Taya-Fokou, Sabine Aimee Touangnou-Chamda, Rachel Audrey Nayang-Mundo, Inès Nyebe, Jeannette Nina Magoudjou-Pekam, Jacqueline Félicité Yéngué, Larissa Gertrude Djukouo, Cynthia Paola Demeni Emoh, Hervé Raoul Tazokong, Arnol Bowo-Ngandji, Eric Lontchi-Yimagou, Afi Leslie Kaiyven, Valerie Flore Donkeng Donfack, Richard Njouom, Jean Claude Mbanya, Wilfred Fon Mbacham, Sara Eyangoh

**Affiliations:** 1 Camdiagnostic, Ministry of Scientific Research and Innovation, Yaounde, Cameroon; 2 Faculty of Medicine and Biomedical Sciences, The University of Yaounde I, Yaounde, Cameroon; 3 Department of Mycobacteriology, Centre Pasteur of Cameroon, Yaounde, Cameroon; 4 Virology Department, Centre Pasteur of Cameroon, Yaounde, Cameroon; 5 Bacteriology Department, Centre Pasteur of Cameroon, Yaounde, Cameroon; 6 Medical Research Centre, Institute of Medical Research and Medicinal Plants Studies, Yaounde, Cameroon; 7 Department of Microbiology, The University of Yaounde I, Yaounde, Cameroon; 8 Evaluation and Research Unit, National AIDS Control Committee, Yaounde, Cameroon; 9 Department of Biochemistry, The University of Yaounde I, Yaounde, Cameroon; 10 Department of Microbiology, Protestant University of Central Africa, Yaounde, Cameroon; 11 Department of Animals Biology and Physiology, The University of Yaounde I, Yaounde, Cameroon; 12 Laboratory for Molecular Medicine and Metabolism, The University of Yaounde I, Yaounde, Cameroon; 13 Institute of Biomedical and Clinical Research, University of Exeter, Exeter, United Kingdom; 14 The Biotechnology Centre, The University of Yaounde I, Yaounde, Cameroon; Universidad Miguel Hernandez de Elche, SPAIN

## Abstract

**Introduction:**

Meta-analyses conducted so far on the association between diabetes mellitus (DM) and the tuberculosis (TB) development risk did not sufficiently take confounders into account in their estimates. The objective of this systematic review was to determine whether DM is associated with an increased risk of developing TB with a sensitivity analyses incorporating a wider range of confounders including age, gender, alcohol consumption, smoke exposure, and other comorbidities.

**Methods:**

Pubmed, Embase, Web of Science and Global Index Medicus were queried from inception until October 2020. Without any restriction to time of study, geographical location, and DM and TB diagnosis approaches, all observational studies that presented data for associations between DM and TB were included. Studies with no abstract or complete text, duplicates, and studies with wrong designs (review, case report, case series, comment on an article, and editorial) or populations were excluded. The odds ratios (OR) and their 95% confidence intervals were estimated by a random-effect model.

**Results:**

The electronic and manual searches yielded 12,796 articles of which 47 were used in our study (23 case control, 14 cross-sectional and 10 cohort studies) involving 503,760 cases (DM or TB patients) and 3,596,845 controls. The size of the combined effect of TB risk in the presence of DM was OR = 2.3, 95% CI = [2.0–2.7], I^2^ = 94.2%. This statistically significant association was maintained in cohort (OR = 2.0, CI 95% = [1.5–2.4], I^2^ = 94.3%), case control (OR = 2.4, CI 95% = [2.0–2.9], I^2^ = 93.0%) and cross-sectional studies (OR = 2.5, CI 95% = [1.8–3.5], I^2^ = 95.2%). The association between DM and TB was also maintained in the sensitivity analysis including only studies with similar proportions of confounders between cases and controls. The substantial heterogeneity observed was mainly explained by the differences between geographic regions.

**Conclusions:**

DM is associated with an increased risk of developing latent and active TB. To further explore the role of DM in the development of TB, more investigations of the biological mechanisms by which DM increases the risk of TB are needed.

**Review registration:**

PROSPERO, CRD42021216815.

## Introduction

About 25% of the global population is infected with Mycobacterium tuberculosis (MTB) [1], including nearly 10 million new cases of active tuberculosis (TB) and 1.5 million deaths recorded each year [2]. These statistics have crowned TB as one of the leading causes of death from infectious diseases worldwide. MTB infections are more prevalent in developing regions of Southeast Asia (44%), Africa (25%) and the West Pacific (18%), with 2/3 of cases recorded in India, Indonesia, China, Philippines, Pakistan, Nigeria, Bangladesh and South Africa [2]. The International Diabetes Federation estimated that nearly half a billion people (about 10% of the global population) were living with diabetes mellitus (DM) each year, including more than 4 million deaths [3]. This incidence is predicted to increase by more than 10% by 2045, leading to about 700 million cases. The majority of people living with DM are registered in the urban areas of low-and middle-income countries where TB is also dominant. Five of 8 countries with the highest incidence of TB are among the 10 countries with the highest prevalence of DM [2, 3].

Compared to patients with TB only, patients with TB and DM are more likely to have more severe clinical pictures, greater infectivity, treatment failure for TB, relapses after recovery, and high mortality [4–8]. The global escalation of DM and TB epidemics is therefore detrimental and especially for low-resource countries where a very high proportion of DM remains undiagnosed or untreated due to poor resourced health systems [9, 10]. This high increase of DM patients in areas with high TB endemicity is of great concern to TB control efforts because numerous studies have suggested that DM increases the risk of developing latent and active TB [11, 12]. Diabetes mellitus is indeed a disease that can alter the host’s immunity and lead to increased susceptibility to several diseases including tuberculosis [13]. The association between DM and TB has been established in several systematic reviews including active TB [14, 15], latent TB [16] and multidrug-resistant TB [17, 18]. There are multiple confounding factors for the association between DM and TB, the main ones being: HIV infections [19, 20], undernutrition [21], smoking and alcoholism [22, 23]. Although all of these reviews have been devoted to the association between DM and TB, apart from adjusting analyses for age [14, 24], other major confounding factors such as HIV infection, alcohol or smoke exposure have received very little attention. In view of the increasing incidence of DM epidemic, further evidence of the association of DM and TB would be of crucial importance in the fight against the double DM-TB epidemic [25]. Furthering this knowledge could include implications such as the implementation of education, prevention, two-way early detection and co-management programs for MD and TB [26]. In this meta-analysis, including a sensitivity analysis with studies with similar proportions of confounders among cases and controls, we further assess the association between DM and TB.

## Methods

### Literature search

Preferred Reporting Items for Systematic Reviews and Meta-Analyses (PRISMA) guidelines were followed for the preparation (PROSPERO ID = CRD42021216815, https://www.crd.york.ac.uk/prospero/display_record.php?ID=CRD42021216815) and writing of this review (S1 Table). A comprehensive search strategy for relevant articles was applied in several electronic databases including Pubmed, Embase, Web of Science, and Global Index Medicus. We searched from the date the databases were created to October 2020. The search terms covered exposure (DM) and outcome (TB) (S2 Table). Beyond this electronic search, we performed an additional review of the bibliographic references of relevant works for additional inclusions.

### Inclusion and non-inclusion criteria

We included in the present review, all observational studies (cohort, case-control and cross-sectional) which investigated the association between DM and TB without any restriction by geographic location, time and DM and TB diagnostic approaches. The studies included were those written in English or French. Excluded from this review were studies for which we did not have access to the abstract and/or full-text, duplicates, studies with designs or populations inappropriate for the purposes of the present work.

### Study selection and data extraction

The results of the manual and electronic search were screened by two investigators (JETB and SK) using the Rayyan review application. Eligibility and data extraction from full texts were carried out by all investigators in this review. The following parameters were extracted from the included articles: first author, year of publication, study design, sampling approach, timing (retrospectively/prospectively) of (exposure follow up, timing of DM and TB testing), country, study period and duration, age range of participants, DM and TB testing approaches, DM and TB case definition, inclusion and exclusion criteria, pairing parameters, data on qualitative and quantitative confounding factors and data on the total numbers of cases (diabetic or TB) and controls. Qualitative confounders included gender, smoking, alcohol consumption, HIV infection, malignant diseases, chronic kidney diseases, and several other socio-demographic and co-morbidities. Quantitative confounders included age, body mass index, and several other blood components. Discussion and consensus among investigators were used if there were any disagreement.

### Quality assessment

The quality of the included observational studies was assessed according to the Joanna Briggs Institute scale (S3 Table) [27]. The cross-sectional, case-control and cohort studies consisted of 8, 10 and 11 questions respectively with the expected answers being (Yes, No, Unclear or Not applicable). We attributed 1 mark for the answers (Yes) and 0 for the other answers (No, Unclear and Not applicable). We rated studies as having low, moderate, and high risk of bias according to total marks per study. All investigators in this study independently collected answers to the Joanna Briggs Institute scale questions in duplicate. Disagreements were resolved by discussion and consensus.

### Statistical analysis

We opted to group the results according to study designs (cross-sectional, control cases, and cohorts). We selected the data from the reference methods (culture for active TB, IGRA for latent TB, and OGTT for DM) in studies reporting multiple data on the relationship between DM and TB for the same population. The odds ratio (OR), its confidence interval (95% CI) and the prediction interval were calculated using random-effects models on the R software version 4.0.3 [28]. Egger’s test (< 0.1) and funnel charts (asymmetric distribution) were used to indicate the existence of publication bias [29]. The Chi-square test and the I2 and H indices were used to estimate heterogeneity in the estimates [30]. Subgroup analyses and metaregression were used to investigate the parameters responsible for the heterogeneity. Parameters included in these subgroup analyses included sampling method, number of study sites, timing of DM and TB testing, country, country income level [31], and study duration. P values < 0.05 indicated statistical significance. Sensitivity analyses that included only studies with a low risk of bias and studies comparable with regard to confounding factors were performed to enhance the accuracy of our results. We determined the comparability of studies with confounding factors using Chi-square, Fisher or Student’s T-test as reported previously [32].

## Results

### Study selection

**Fig 1:**
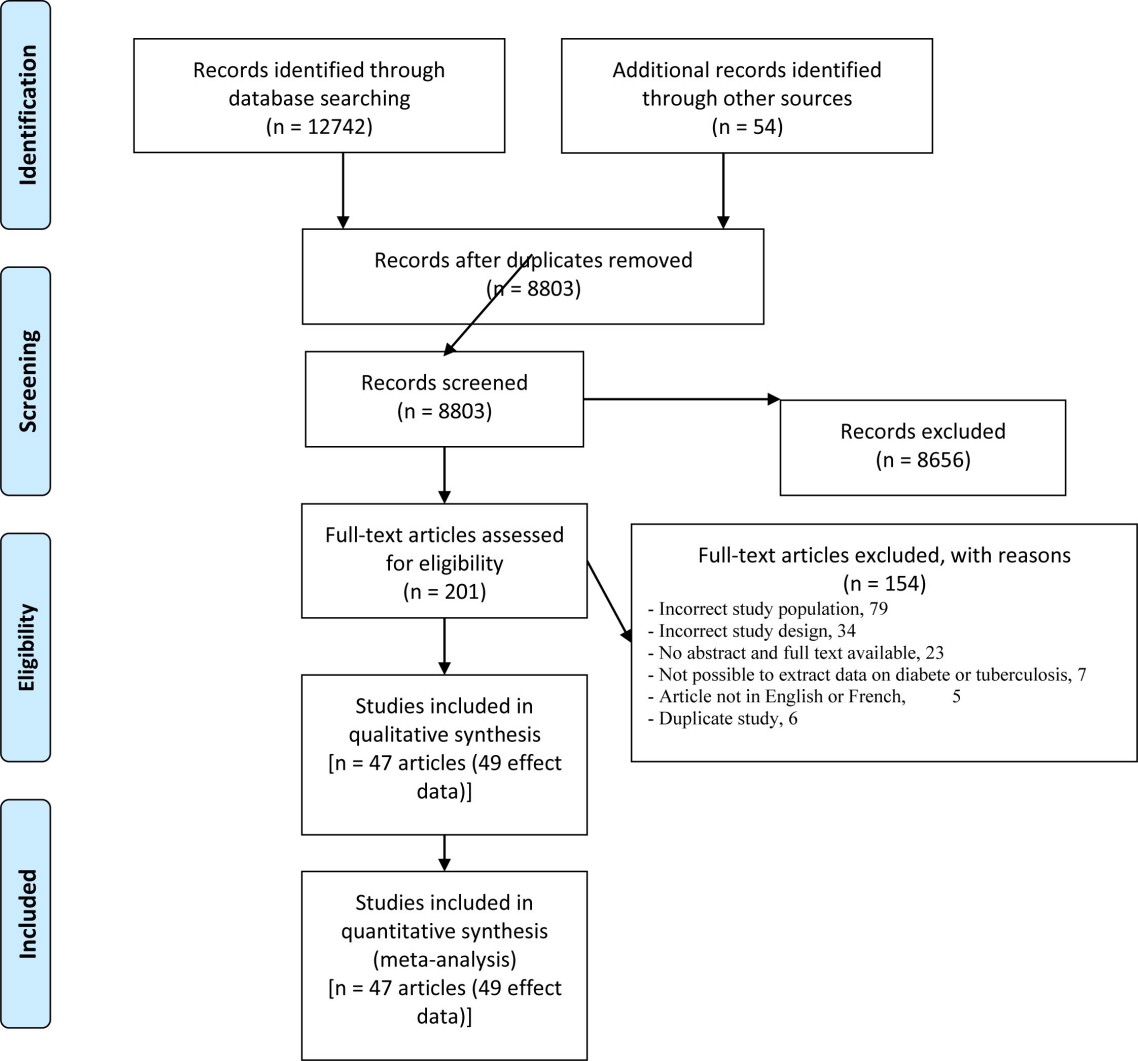
PRISMA flow-chart of studies selected for the meta-analysis.

The electronic search yielded 12,742 articles from PubMed (6725), Web of Science (6123), Embase (693), and Global Index Medicus (201). Manual search yielded 54 additional articles (Fig 1). From these, the eligibility review resulted in 201 articles and the exclusion brought this number to 154 (S4 Table) and finally the inclusion resulted 47 articles used (49 effect estimates) in this review [33–79].

### Summary characteristics of included studies

The detailed description of the individual characteristics of the included studies is presented in Table 1. The included studies were published between 1992 and 2020. Cases (TB), controls (non-TB), exposed (diabetics) and unexposed (non-diabetics) were recruited from 1976 to 2018. The majority of studies had a case-control design (23/49), while 16 were cross-sectional and 10 cohort studies. No investigator of the included studies performed a prospective follow-up of exposed/unexposed subjects in the included studies. Five studies were representative of the national population. Included studies were performed in 18 countries spread across different regions of the world and more particularly in China (16/49) and the United States of America (11/49). High-income countries (26/49) were the most represented and only one study was conducted in low-income countries [46]. The vast majority of studies recruited adults over 15 years old. Apart from studies with multiple diagnostic methods, the International Classification of Diseases (ICD) code was the most widely used approach for DM (10/26) and TB (11/20). Nineteen included studies paired reference with controls with at least one parameter.

**Table 1:** Individual characteristics of included studies.

Study Design	Country	Study period	TB stage	TB diagnosis approach	DM diagnosis approach	Controls	Matched parameters between cases and controls	Author, Year
Case control	Indonesia	Mar/2001-Mar/2005	Active TB	Clinical, chest X-rays, Microscopy	Fasting blood glucose	Presumed healthy controls	Age, Gender, county of residence	Alisjahbana, 2006 [33]
Cross sectional	China	Aug/2001-Dec/2004	Active TB	ICD code, Medical records	ICD code, Medical records	Non-DM	Unclear/ Not reported	Baker, 2012 [34]
Cross sectional	United States of America	2011–2012	Latent TB infection	IGRA Test, Tuberculin skin test	Doctor-diagnosed DM, Glycated hemoglobin A1c test	Non-TB diseases	Unclear/ Not reported	Barron, 2018 –DM [35]
Cross sectional	United States of America	2011–2012	Latent TB infection	IGRA Test, Tuberculin skin test	Doctor-diagnosed DM, Glycated hemoglobin A1c test	Non-TB diseases	Unclear/ Not reported	Barron, 2018 –PreDM [35]
Case control	Tanzania	Jun/2012-Dec/2013	Active TB	Clinical, chest X-rays, Microscopy	Fasting blood glucose, Oral glucose tolerance test, Glycated hemoglobin A1c test	Presumed healthy controls	Age, Gender	Boillat-blanco, 2016 [36]
Case control	United States of America	Sep/1998-Dec/2003	Active TB	ICD code	ICD code	Presumed healthy controls	Unclear/ Not reported	Brassard, 2006 [37]
Case control	United States of America	1988–1990	Active TB	Microscopy, Culture, PCR	Unclear/ Not reported	Non-TB diseases	Unclear/ Not reported	Buskin, 1994 [38]
Cross sectional	China	Jan/1983- Dec/2003	Active TB	Clinical, chest X-rays, Culture	Unclear/ Not reported	Non-TB diseases	Unclear/ Not reported	Chen, 2006 [39]
Case control	China	1997–2010	Active TB	ICD code	ICD code	Presumed healthy controls	Age, Gender, Recruitment time	Chung, 2014 [40]
Case control	United States of America	1976–1980	Active TB	Doctor-diagnosed TB	Doctor-diagnosed DM, Oral glucose tolerance test	Non-TB diseases	Unclear/ Not reported	Corris, 2012 [41]
Case control	Kazakhstan	Jun/ 2012- May/ 2014	Active TB	Clinical, chest X-rays, Culture	Doctor-diagnosed DM	Presumed healthy controls	County of residence	Davis, 2017 [42]
Case control	Tanzania	Apr/2006-Jan/2009	Active TB	Microscopy, Culture	Fasting blood glucose, Oral glucose tolerance test	Presumed healthy controls	Age, Gender	Faurholt-Jepsen, 2011 [44]
Cross sectional	Tanzania	Apr/ 2006—Mar/ 2009	Active TB	Culture	Fasting blood glucose, Oral glucose tolerance test	Presumed healthy controls	Age, Gender	Faurholt-Jepsen, 2014 [43]
Cohort	United States of America	Jan/ 2001—Dec/ 2011	Active TB	chest X-rays	ICD code, Fasting blood glucose	Non-DM	Unclear/ Not reported	Golub, 2019 [45]
Case control	Guinea-Bissau	July/2010-July/2011	Active TB	Clinical, chest X-rays, Microscopy	Fasting blood glucose, Random blood sugar test	Presumed healthy controls	Unclear/ Not reported	Haraldsdottir, 2015 [46]
Cross sectional	United States of America	October/2013-August/2014	Latent TB infection	chest X-rays, IGRA Test	Glycated hemoglobin A1c test	Non-TB diseases	Unclear/ Not reported	Hensel, 2016 [47]
Case control	Bangladesh	Jan/2008-Jul/2008	Active TB	Microscopy	Oral glucose tolerance test	Non-TB diseases	Unclear/ Not reported	Hossain, 2014 [48]
Case control	United Kingdom	1990–2001	Active TB	Medical records	Medical records	Presumed healthy controls	Age, Gender, County of residence	Jick, 2006 [49]
Case control	Croatia	2006–2008	Active TB	Culture	Unclear/ Not reported	Non-TB diseases	Age, Gender, county of residence	Jurcev-Savicevic, 2013 [50]
Cross sectional	Thailand	Mar/2012-Mar/2013	Latent TB infection	Tuberculin skin test, IGRA Test	Unclear/ Not reported	Presumed healthy controls	Unclear/ Not reported	Khawcharoenporn, 2015 [51]
Cohort	Korean	1988–1990	Active TB	chest X-rays, Microscopy, Culture	Glucose oxidase test	Non-DM	Unclear/ Not reported	Kim, 1995 [52]
Cross sectional	India	May/2014-Nov/2015	Active TB	Tuberculin skin test, Microscopy, Culture	Clinical, Random blood sugar test	Presumed healthy controls	Unclear/ Not reported	Kubiak, 2019 [53]
Cohort	China	2000–2011	Active TB	ICD code	ICD code	Presumed healthy controls	Age, Gender	Kuo, 2013 [54]
Case control	China	1998–2011	Active TB	ICD code	ICD code	Non-TB diseases	Age, Gender	Lai, 2014 [55]
Cohort	China	1997–2007	Active TB	ICD code	ICD code	Non-DM	Age, Gender, Recruitment time	Lee, 2013 [56]
Case control	China	2006–2017	Active TB	Clinical, Medical records, chest X-rays, Microscopy, Culture	ICD code, Medical records, Fasting blood glucose, Glycated hemoglobin A1c test	Non-TB diseases	Unclear/ Not reported	Lee, 2014 [58]
Cohort	China	Mar/2005-Dec/2012	Active TB	ICD code, Medical records	Fasting blood glucose	Non-DM	Unclear/ Not reported	Lee, 2016 [57]
Case control	Denmark	Jan/1980-Dec/2008	Active TB	ICD code	Clinical, Medical records, Glycated hemoglobin A1c test	Non-TB diseases	Age, Gender, county of residence	Leegaard, 2011 [59]
Cohort	China	Jan/2000—Dec/2005	Active TB	Clinical, Medical records, chest X-rays, Histopathology, Culture	Glycated hemoglobin A1c test	Non-DM	Unclear/ Not reported	Leung, 2008 [60]
Cohort	China	2000–2009	Active TB	ICD code	ICD code	Non-DM	Age, Gender, Recruitment time	Lin, 2017 [62]
Cohort	China	2005–2013	Latent TB infection	Clinical, chest X-rays, Tuberculin skin test, IGRA Test	Doctor-diagnosed DM	Non-DM	Unclear/ Not reported	Lin, 2019 [61]
Case control	India	Jan/1983-Dec/1989	Active TB	Tuberculin skin test	Medical records, Fasting blood glucose, Any glucose level	Non-TB diseases	Unclear/ Not reported	Mori, 1992 [63]
Case control	Romania	Mar/2014—Mar/2015	Active TB	Microscopy, Culture, PCR	Unclear/ Not reported	Non-TB diseases	Age, Gender, county of residence	Ndishimye, 2017 [64]
Case control	United States of America	1991	Active TB	ICD code	ICD code	Non-TB diseases	Unclear/ Not reported	Pablos-Méndez, 1997 [65]
Cohort	United Kingdom	Jan/1990-Dec/2012	Active TB	ICD code	ICD code	Non-DM	Age, Gender	Pealing, 2015 [66]
Case control	Brazil	Aug/2008-Apr/2010	Active TB	Clinical, Microscopy, Culture	Fasting blood glucose, Oral glucose tolerance test	Non-TB diseases	Age, Gender	Pereira, 2016 [67]
Case control	United States of America	1999–2001	Active TB	ICD code	ICD code	Non-TB diseases	Unclear/ Not reported	Pérez, 2006 [68]
Cohort	China	2002–2011	Active TB	ICD code	ICD code	Non-DM	Gender	Shen, 2014 [69]
Case control	Japan	Jan/2015-Dec/2018	Active TB	Clinical, chest X-rays, IGRA Test, Microscopy, Culture, PCR	Doctor-diagnosed DM	Non-TB diseases	County of residence	Shimouchi, 2020 [70]
Cross sectional	China	Mar/2011-Feb/2012	Latent TB infection	ELISA, Microscopy, Culture	Unclear/ Not reported	Non-TB diseases	Unclear/ Not reported	Shu, 2012 [71]
Cross sectional	United States of America	Apr/2005-Mar/2012	Latent TB infection	Tuberculin skin test, IGRA Test	Medical records	Non-DM	Unclear/ Not reported	Suwanpimolkul, 2014 [72]
Cross sectional	United States of America	Apr/2005-Mar/2012	Active TB	Tuberculin skin test, IGRA Test	Medical records	Non-DM	Unclear/ Not reported	Suwanpimolkul, 2014 [72]
Cross sectional	Malaysia	Oct/2014-Dec/2015	Latent TB infection	Clinical, chest X-rays, Tuberculin skin test, Microscopy	Fasting blood glucose, Glycated hemoglobin A1c test, Random blood sugar test	Non-DM	Unclear/ Not reported	Swarna Nantha, 2017 [73]
Cross sectional	China	Jan/2011-Dec/2012	Latent TB infection	Medical records, chest X-rays, IGRA Test	Unclear/ Not reported	Non-TB diseases	Unclear/ Not reported	Ting, 2014 [74]
Case control	Republic of Kiribati	Jun/2010-Mar/2012	Latent TB infection	Clinical, Doctor-diagnosed DM, chest X-rays, Tuberculin skin test, Microscopy, Culture	Glycated hemoglobin A1c test	Non-TB diseases	Unclear/ Not reported	Viney, 2015 [75]
Cross sectional	China	Sep/2010-Dec/2012	Active TB	Clinical, chest X-rays, Microscopy	Fasting blood glucose	Non-TB diseases	County of residence	Wang, 2013 [76]
Cross sectional	Indonesia	2014–2015	Active TB	Doctor-diagnosed DM	Doctor-diagnosed DM	Non-TB diseases	Unclear/ Not reported	Wardhani, 2019 [77]
Cross sectional	China	Jan/2002-Dec/2004	Active TB	Culture	Medical records	Presumed healthy controls	Unclear/ Not reported	Wu, 2007 [78]
Case control	Kazakhstan	Jun/2012-Jan/2013	Active TB	Clinical, chest X-rays, Microscopy, Culture, PCR	Unclear/ Not reported	Presumed healthy controls	Age	Zhussupov, 2016 [79]

DM: Diabetes Mellitus; ICD: International Classification of Diseases; TB: Tuberculosis.

### Risk of bias in included studies

The methodological quality of the included studies is shown in S5 Table. Overall, the included studies had a low risk of bias (32/49). Most of the included studies collected data and considered confounding factors in the analysis of the association between DM and the TB development risk. In cohort studies, diabetic and nondiabetic patients were generally recruited from the same population, diagnosed with DM and TB in the same way, tested for absence of TB at the start of the follow-up, and followed up with a completeness rate. TB and non-TB patients recruited from case control studies were generally comparable and diagnosed with TB and DM in the same way. In cross-sectional studies, the study context and inclusion criteria for participants were well defined.

### Meta-analysis

**Fig 2:**
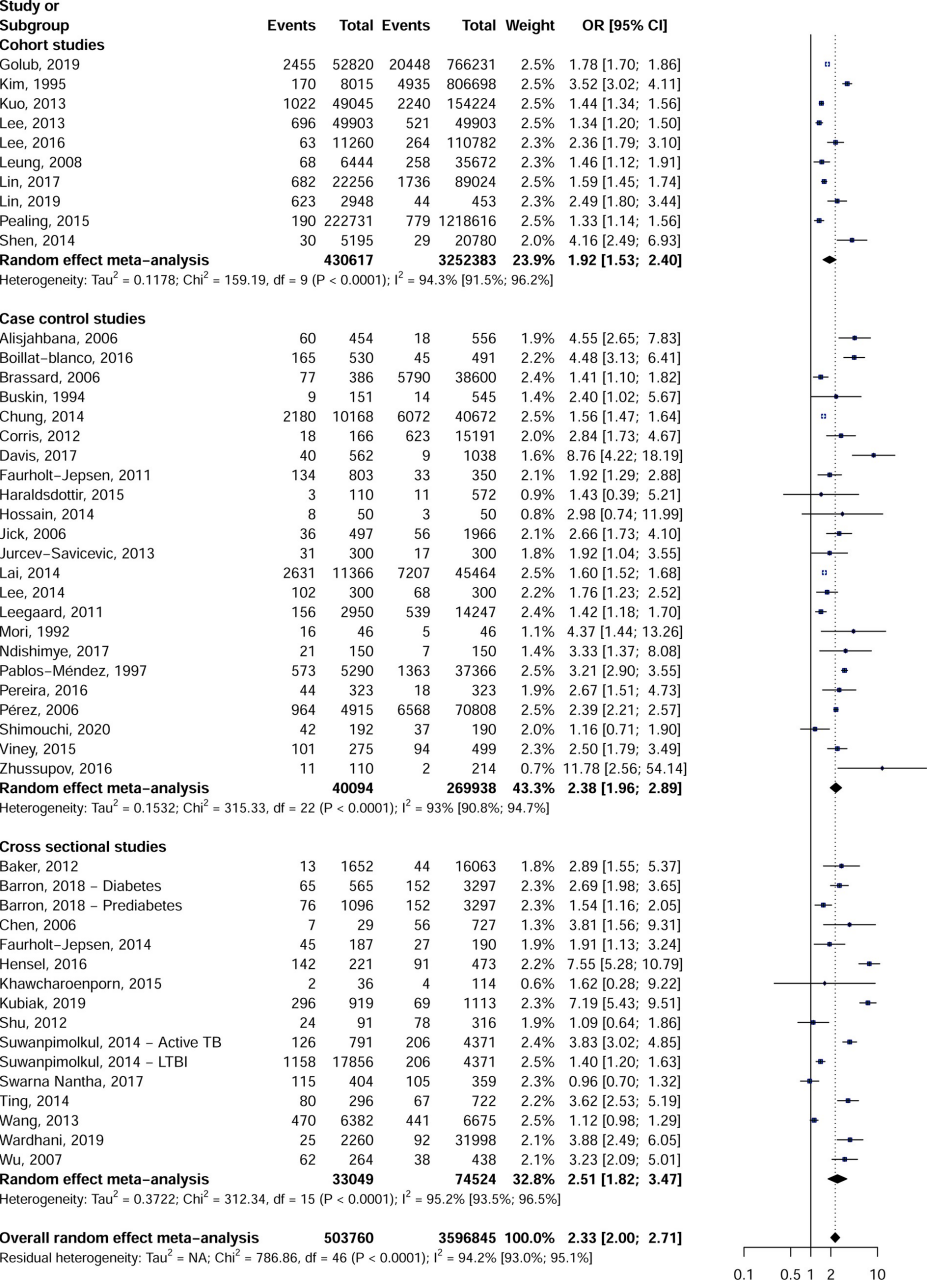
Association between diabetes mellitus and risk of tuberculosis in cohort, case control and cross-sectional studies.

In this meta-analysis, 503,760 cases (diabetic or TB) and 3,596,845 controls were considered to calculate the combined effect of the association between DM and the TB risk. Regarding the study design, the 49-effect estimate showed an increased risk of developing TB in diabetic patients (OR = 2.3, 95% CI = [2.0–2.7]) (Fig 2). This overall effect was associated with substantial heterogeneity (I2 = 94.2% [93.0–95.1]). The association between DM and the risk of developing TB was conserved in the 10 cohort (OR = 2.0, CI 95% = [1.5–2.4]), the 23 case-control (OR = 2.4, CI 95% = [2.0–2.9]) and 16 cross-sectional studies (OR = 2.5, CI 95% = [1.8–3.5]). A significant publication bias was recorded in the cross-sectional (p Egger = 0.058) and the case-control studies (p Egger = 0.093) unlike the cohort studies (p Egger = 0.417) which did not present any publication bias (Table 2, S1–S3 Figs). Considering only studies with low risk of bias sensitivity analysis did not reveal any difference from the overall results. The data collected for 81 qualitative variables and 16 quantitative variables considered to be confounding factors enabled us to select studies that had similar proportions in references and controls (S6 and S7 Tables). For cohort studies, sensitivity analyses including only comparable studies for confounding factors showed similar results to overall results, including factors such as HIV infection, malignancies and age. For the case-control studies, the same trend was observed for the sensitivity analysis including only comparable studies mainly for alcohol drinkers, chronic kidney disease, drug users, HIV infected patients, tobacco exposure, and age. For the cross-sectional studies, on the other hand, the overall effect observed was lost for the sensitivity analyses including only comparable studies for certain confounding factors including chronic kidney disease, patients with cirrhosis of the liver or malignant diseases.

**Table 2:** TB development in people with and without DM and influence of confounders.

	OR (95%CI)	95% Prediction interval	N Studies	N LRTI cases	N controls	H (95%CI)	I^2^ (95%CI)	P heterogeneity	P Egger test
**Cohort studies**									
Overall	1.9 [1.5–2.4]	[0.8–4.4]	10	430617	3252383	4.2 [3.4–5.2]	94.3 [91.5–96.2]	< 0.001	0.417
Low risk of bias	1.6 [1.4–1.7]	[1.1–2.2]	7	414459	2424452	2.9 [2.1–4]	88.2 [78.2–93.7]	< 0.001	0.496
Asbestosis	1.6 [1.5–1.7]	NA	1	22256	89024	NA	NA	1	NA
Autoimmune disorders	1.3 [1.2–1.5]	NA	1	49903	49903	NA	NA	1	NA
Bet nut use	2.4 [1.8–3.1]	NA	1	11260	110782	NA	NA	1	NA
Chronic kidney disease	1.8 [1.7–1.9]	NA	1	52820	766231	NA	NA	1	NA
HIV infection	1.5 [1.3–1.7]	NA	2	72159	138927	2.3 [1.1–4.7]	81 [19.2–95.6]	0.022	NA
Male gender	1.8 [1.2–2.5]	[0.3–9.4]	4	83798	195379	2.6 [1.7–4.1]	85.4 [63.9–94.1]	< 0.001	0.377
Malignant disease	1.8 [1.7–1.8]	NA	2	59264	801903	1.4	49	0.161	NA
Pneumoconiosis	1.6 [1.5–1.7]	NA	1	22256	89024	NA	NA	1	NA
Age	1.5 [1.3–1.7]	NA	2	72159	138927	2.3 [1.1–4.7]	81 [19.2–95.6]	0.022	NA
**Case control studies**									
Overall	2.4 [2–2.9]	[1–5.5]	23	40094	269938	3.8 [3.3–4.4]	93 [90.8–94.7]	< 0.001	0.093
Low risk of bias	2.2 [1.7–2.9]	[0.8–6.2]	13	28831	144497	2.6 [2.1–3.3]	85.4 [76.6–90.9]	< 0.001	0.05
Adenotonsillectomy	1.6 [1.5–1.7]	NA	1	11366	45464	NA	NA	1	NA
Central sewage system	1.9 [1–3.5]	NA	1	300	300	NA	NA	1	NA
Chronic kidney disease	1.9 [1.4–2.6]	[0.3–14]	3	710	814	1.7 [1–3.1]	64.7 [0–89.9]	0.059	0.271
Co_morbidity	1.9 [1–3.5]	NA	1	300	300	NA	NA	1	NA
Currently rent home	11.8 [2.6–54.1]	NA	1	110	214	NA	NA	1	NA
Drinker	3.2 [2.9–3.5]	[2.5–3.9]	4	5850	38388	1.2 [1–1.9]	26.3 [0–72.1]	0.254	0.101
Drug user	3.8 [1.8–7.9]	[0–16592.3]	3	1012	1488	2.2 [1.2–3.9]	79.5 [34.7–93.5]	0.008	0.916
Ever injected heroin	8.8 [4.2–18.2]	NA	1	562	1038	NA	NA	1	NA
Ever smoked heroin	8.8 [4.2–18.2]	NA	1	562	1038	NA	NA	1	NA
Ever used opium	8.8 [4.2–18.2]	NA	1	562	1038	NA	NA	1	NA
Extra pulmonary lesion	1.8 [1.2–2.5]	NA	1	300	300	NA	NA	1	NA
Family history of diabetes mellitus	3 [0.7–12]	NA	1	50	50	NA	NA	1	NA
Hepatitis C infection, Anti_HCV	11.8 [2.6–54.1]	NA	1	110	214	NA	NA	1	NA
HIV infection	1.5 [1.3–1.8]	[0.5–4.6]	3	3360	14761	2 [1.1–3.6]	74.9 [16.8–92.4]	0.019	0.277
Hyperlipidaemia	1.6 [1.5–1.6]	NA	1	10168	40672	NA	NA	1	NA
Illicit drug use	1.9 [1–3.5]	NA	1	300	300	NA	NA	1	NA
Immunosuppressive therapy	1.9 [1–3.5]	NA	1	300	300	NA	NA	1	NA
Living in a crowded home	4.6 [2.6–7.8]	NA	1	454	556	NA	NA	1	NA
Male gender	2.1 [1.6–2.7]	[0.9–4.9]	10	26090	117338	2.1 [1.5–2.8]	77.2 [58.2–87.6]	< 0.001	0.036
Malignant disease	1.8 [1.2–2.5]	NA	1	300	300	NA	NA	1	NA
Marital status, Single	2.1 [1.5–3.1]	NA	2	953	500	1.1	17.3	0.272	NA
Other chronic diseases	1.9 [1–3.5]	NA	1	300	300	NA	NA	1	NA
Pancreatitis	2.4 [1–5.7]	NA	1	151	545	NA	NA	1	NA
Physical activity	3 [0.7–12]	NA	1	50	50	NA	NA	1	NA
Poly_drug resistant	1.8 [1.2–2.5]	NA	1	300	300	NA	NA	1	NA
Previous hospitalizations	1.9 [1–3.5]	NA	1	300	300	NA	NA	1	NA
Prisoners	1.9 [1–3.5]	NA	1	300	300	NA	NA	1	NA
Smoke Exposure	2.5 [2–3.3]	[1.4–4.4]	4	702	16807	1 [1–1.5]	0 [0–53.1]	0.806	0.86
Transplantation	1.9 [1–3.5]	NA	1	300	300	NA	NA	1	NA
Age	2.4 [1.6–3.7]	[0.6–10.1]	5	4756	15561	2.9 [2–4.3]	88.5 [75.8–94.5]	< 0.001	0.393
Cigarettes smoked in a week	8.8 [4.2–18.2]	NA	1	562	1038	NA	NA	1	NA
**Cross sectional studies**									
Overall	2.5 [1.8–3.5]	[0.6–9.7]	16	33049	74524	4.6 [3.9–5.3]	95.2 [93.5–96.5]	< 0.001	0.058
Low risk of bias	2.4 [1.6–3.5]	[0.5–11.4]	12	30309	41171	5.2 [4.4–6.1]	96.3 [94.8–97.3]	< 0.001	0.15
Anemia	1.1 [0.6–1.9]	NA	1	91	316	NA	NA	1	NA
Atrial fibrillation	1 [0.7–1.3]	NA	1	404	359	NA	NA	1	NA
Autoimmune disorders	2.1 [0.9–4.7]	NA	2	387	1038	3.7 [2–6.7]	92.5 [74.7–97.8]	< 0.001	NA
Bronchial asthma	1 [0.7–1.3]	NA	1	404	359	NA	NA	1	NA
Bronchiectasis	3.2 [2.1–5]	NA	1	264	438	NA	NA	1	NA
Chronic kidney disease	1.9 [0.7–4.6]	NA	2	700	1081	5.4 [3.3–8.9]	96.6 [90.9–98.7]	< 0.001	NA
Chronic liver disease	1 [0.7–1.3]	NA	1	404	359	NA	NA	1	NA
Chronic obstructive pulmonary disease	1 [0.7–1.3]	NA	1	404	359	NA	NA	1	NA
Drinker	5 [2.6–9.6]	NA	2	1873	16536	2.6 [1.3–5.3]	85.5 [41.6–96.4]	0.009	NA
Gout	1 [0.7–1.3]	NA	1	404	359	NA	NA	1	NA
Health care worker	3.6 [2.5–5.2]	NA	1	296	722	NA	NA	1	NA
Hemodialysis patients	1.9 [0.9–4.1]	NA	2	355	754	3.1 [1.6–5.9]	89.5 [60.9–97.2]	0.002	NA
Hepatitis B infection, HBsAg	2.5 [1.3–4.8]	[0.2–29.5]	5	2315	8153	4.5 [3.4–6]	95.1 [91.2–97.3]	< 0.001	0.597
Hepatitis C infection, Anti_HCV	3.5 [1.5–7.8]	[0–71340.3]	3	1346	4497	4.8 [3.3–7.1]	95.7 [90.7–98]	< 0.001	0.811
HIV infection	5.2 [3.1–8.7]	NA	2	517	1195	2.8 [1.4–5.6]	87.6 [51.8–96.8]	0.005	NA
Ischaemic heart disease	1 [0.7–1.3]	NA	1	404	359	NA	NA	1	NA
Liver cirrhosis	2.1 [0.9–4.7]	NA	2	387	1038	3.7 [2–6.7]	92.5 [74.7–97.8]	< 0.001	NA
Living in a crowded home	2.9 [1.6–5.4]	NA	1	1652	16063	NA	NA	1	NA
Male gender	2.5 [1.6–4]	[0.5–11.9]	7	3841	24363	3.1 [2.3–4.2]	89.9 [81.7–94.4]	< 0.001	0.883
Malignant disease	2.6 [1.6–4.3]	[0.3–22.7]	4	680	2203	2.2 [1.4–3.6]	79.8 [46.4–92.4]	0.002	0.962
Osteoarthritis	1 [0.7–1.3]	NA	1	404	359	NA	NA	1	NA
Residence in an indigenous community	2.9 [1.6–5.4]	NA	1	1652	16063	NA	NA	1	NA
Self_reported history of renal failure	7.2 [5.4–9.5]	NA	1	919	1113	NA	NA	1	NA
Smoke Exposure	3 [1.8–5.2]	[0.4–22.9]	5	2825	20871	3.1 [2.2–4.5]	89.8 [79–95]	< 0.001	0.467
Syphilis	7.5 [5.3–10.8]	NA	1	221	473	NA	NA	1	NA
Thyroid disorder	1 [0.7–1.3]	NA	1	404	359	NA	NA	1	NA
Total Bilirubin (mg_dL), Not Normal	2 [1.4–3]	NA	2	1661	6594	2.6 [1.3–5.3]	85.4 [40.9–96.4]	0.009	NA
Age	2.3 [1.5–3.6]	NA	2	216	917	1.3	41.1	0.193	NA
Body mass index	7.5 [5.3–10.8]	NA	1	221	473	NA	NA	1	NA
Dialysis duration	1.8 [0.8–4.2]	NA	2	120	1043	2.4 [1.1–4.8]	82 [23.9–95.7]	0.018	NA
Hemoglobin	1.1 [1–1.3]	NA	1	6382	6675	NA	NA	1	NA

### Source of heterogeneity examination

The potential sources of heterogeneity were explored by the subgroup analyses. These sources included country, UNSD region, country income level, TB stage (active vs latent), and type of controls (S8 Table). In the cohort, control and cross-sectional designs only the geographic location (countries and UNSD regions) contributed to a source of heterogeneity (p subgroup difference <0.05). In cohort studies, however, all subcategories showed an association between DM and the risk of developing TB.

## Discussion

This systematic review included 47 articles examining the association between DM and TB. Regardless of study design, region of origin, stage of TB (latent or active TB), type of controls (non-DM, non-TB, or presumed healthy), this meta-analysis suggests that DM increases the risk of developing TB. The overall effect observed suggests that patients with DM are two times more likely to develop TB than non-diabetics. This overall effect persisted in the sensitivity analysis including only studies with similar proportions of common confounders between cases and controls.

The statistically significant association between DM and TB observed in this review is consistent with those reported previously. A first qualitative review in 2007 with 9 included studies reported effect estimates ranging from 1.5 to 7.8 fold the risk of TB in DM patients [80]. Two other meta-analyses that included studies with patients with active TB and whose age-adjusted estimates were reported in 2008 and 2018 [14, 24]. One of these meta-analyses reported an estimated 3.1-fold effect for 3 cohort studies and the second an estimated 1.5-fold effect for 14 studies with low risk of bias. A final meta-analysis with studies recruiting patients with latent TB revealed no significant association for one cohort study and a weak association for 12 cross-sectional studies [16]. Compared to these previous systematic reviews, we included over 10 additional articles and used a very rigorous methodology including calculating effect estimates of primary data from included studies and taking into account a wide range of confounding factors of the association between DM and TB listed in the articles included [35, 43, 45, 48, 53, 58, 61, 62, 64, 67, 70, 73, 77, 79]. Little is known about the biological mechanisms that underlie a high risk of developing TB in patients with DM. Several hypotheses linked to an alteration of immune function in diabetics have however been suggested to explain this association between DM and TB [81–84]. These hypotheses include, but not limited to: depressed cellular immunity, alveolar macrophage dysfunction, low levels of interferon gamma, reduction of interleukin-12, and micronutrient deficiency. We recognize several potential limitations to this review. In addition to the fact that most of the included studies used multiple diagnostic approaches for TB and DM, other diagnostic methods including ICD codes, medical records and self-reported data may be associated with some inaccuracies. Different risk factors have been reported for pulmonary TB compared to extra-pulmonary TB. Very few included studies, however, differentiated pulmonary TB from extrapulmonary TB [85, 86]. Similarly, very few included studies reported information on DM types (1 or 2 and pre-DM or DM) and participant glycaemic control. However, these are conditions that influence susceptibility to TB [87]. Very few included studies reported treatment status for participant for TB. Normalization of glycaemic status has been established for TB patients receiving treatment [88, 89]. This could therefore have been the cause of the misclassification of cases and controls in the included studies. The above limitations would justify the substantial heterogeneity recorded in this meta-analysis. As previously reported [90], very few studies included in this meta-analysis were from Africa, thus compromising the generalizability of these results globally. It should also be noted that Africa has the highest rate of undiagnosed DM in the world and may therefore have a specific profile of the association between DM and TB [91].

Due to the inclusion of only observational studies in this meta-analysis, a causal link between DM and the risk of TB cannot be suggested. However, the results of this meta-analysis further strengthen the level of evidence for the association between DM and the risk of TB development. We therefore encourage specific studies on the association between DM and TB in the context of Africa. We advocated public health programs to prevent DM such as strengthening education on risk factors for DM and physical activities and sports. Patients with DM only and healthcare professionals should be educated about their increased risk of active or latent TB development. Two-way screening and management programs for DM and TB including latent TB would help reduce the incidence and burden associated with this double epidemic. Interventional studies to demonstrate the causal link between DM and TB are needed in the future. Further research on the biological mechanism by which DM increases the risk of TB are needed.

## Supporting information

S1 Fig Funnel chart for publications of the association between diabetes and tuberculosis in cohort studies. (PDF)

S2 Fig Funnel chart for publications of the association between diabetes and tuberculosis in case control studies. (PDF)

S3 Fig Funnel chart for publications of the association between diabetes and tuberculosis in cross-sectional studies. (PDF)

S1 Table Preferred reporting items for systematic reviews and meta-analyses checklist. (PDF)

S2 Table Search strategy in Pubmed. (PDF)

S3 Table Items for risk of bias assessment. (PDF)

S4 Table Main reasons of non-inclusion of eligible studies. (PDF)

S5 Table Risk of bias assessment. (PDF)

S6 Table P-value of Khi-2 and Fisher exact tests for qualitative confounding factors. (PDF)

S7 Table P-value of Student test for quantitative confounding factors. (PDF)

S8 Table Subgroup analyses of the association between diabetes and tuberculosis. (PDF)
