# Supplementary material for: Diabetes mellitus and tuberculosis, a systematic review and meta-analysis with sensitivity analysis for studies comparable for confounders
Source: PLoS One. 2021 Dec 10;16(12):e0261246. doi: 10.1371/journal.pone.0261246 (PMC8664214; doi:10.1371/journal.pone.0261246)

S2 Fig. Funnel chart for publications of the association between diabetes and tuberculosis in case control studies.

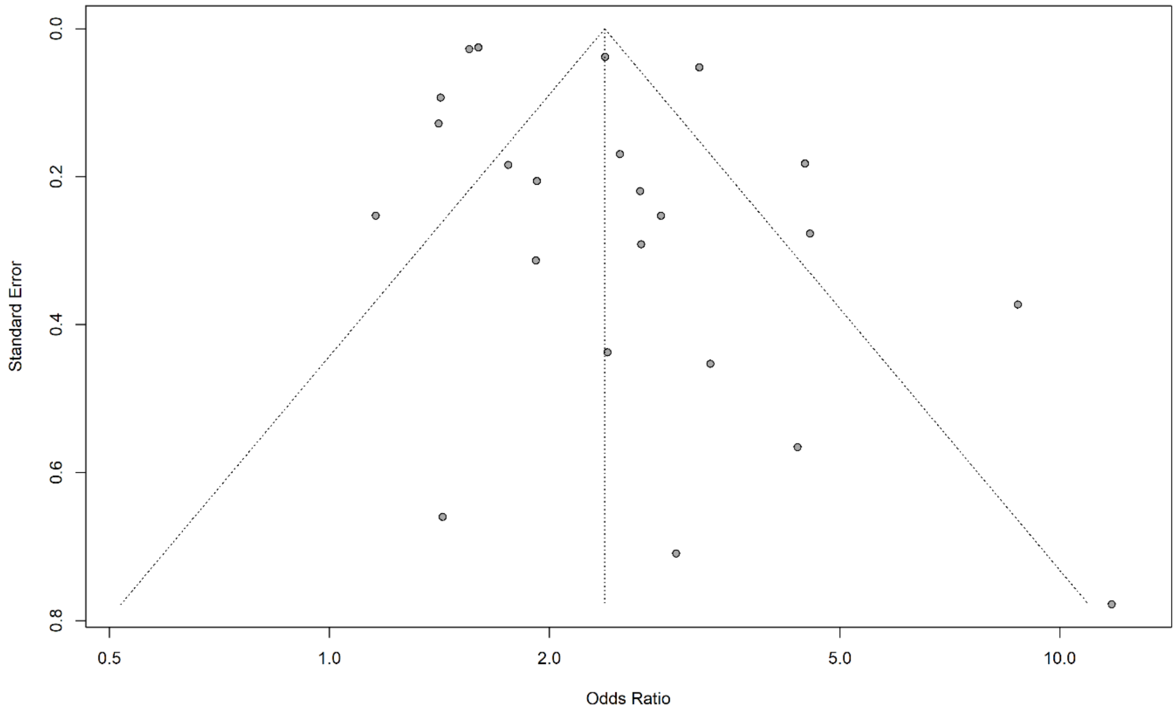

Supplement: S2 Fig — (PDF) [file pone.0261246.s002.pdf]
