# Supplementary material for: Diabetes mellitus and tuberculosis, a systematic review and meta-analysis with sensitivity analysis for studies comparable for confounders
Source: PLoS One. 2021 Dec 10;16(12):e0261246. doi: 10.1371/journal.pone.0261246 (PMC8664214; doi:10.1371/journal.pone.0261246)
Supplement: S2 Table — (PDF) [file pone.0261246.s005.pdf]

S2 Table: Search strategy In Pubmed

Search		Search terms	Items found
#1	Outcomes	Tuberculosis OR Tubercul* OR TB OR Latent Tuberculosis OR Multidrug-Resistant Tuberculosis OR antitubercul* OR MDR-TB OR Mycobacterium tuberculosis OR Mycobacterium tuberculosis Infection OR TB OR koch's disease OR koch disease	368,051
#2	Exposure	Diabetes OR Diabetes Mellitus OR Diabet* OR “insulin resistance” OR T2DM OR T1DM OR Non-insulin-dependent diabetes mellitus OR NIDDM OR IDDM OR Insulin-Dependent Diabetes Mellitus OR hyperglyce* OR hyperlycae* OR glucose toleran* OR glucose intoleran* OR glycemi* OR glycaemi*	879,956
#3		#1 AND #2	7,014
#4		Limit #3 in English and French languages	5,819
