# Supplementary material for: Diabetes mellitus and tuberculosis, a systematic review and meta-analysis with sensitivity analysis for studies comparable for confounders
Source: PLoS One. 2021 Dec 10;16(12):e0261246. doi: 10.1371/journal.pone.0261246 (PMC8664214; doi:10.1371/journal.pone.0261246)
Supplement: S3 Table — (PDF) [file pone.0261246.s006.pdf]

S3 Table. Items for risk of bias assessment

	**The Joanna Briggs Institute Scale for**		
	**cohort studies**	**case control studies**	**cross sectional studies**
	1) Were the DM+ and DM- groups similar and recruited from the same population?	1) Were the TB+ and TB- groups comparable other than presence of TB in cases or absence of TB in controls?	1) Were the criteria for inclusion in the sample clearly defined?
	2) Were the DM measured similarly to assign people to both DM+ and DM- groups?	2) Were TB+ and TB- matched appropriately?	2) Were the study subjects and the setting described in detail?
	3) Was the DM measured in a valid and reliable way?	3) Were the same criteria used for identification of TB+ and TB-?	3) Was the DM measured in a valid and reliable way?
	4) Were confounding factors identified?	4) Was DM measured in a standard, valid and reliable way?	4) Were objective, standard criteria used for measurement of the condition?
	5) Were strategies to deal with confounding factors stated?	5) Was DM measured in the same way for TB+ and TB-?	5) Were confounding factors identified?
	6) Were the DM+ and DM- free of the TB at the start of the study (or at the moment of DM)?	6) Were confounding factors identified?	6) Were strategies to deal with confounding factors stated?
	7) Were the TB measured in a valid and reliable way?	7) Were strategies to deal with confounding factors stated?	7) Were the TB measured in a valid and reliable way?
	8) Was the follow up time reported and sufficient to be long enough for TB to occur?	8) Were TB assessed in a standard, valid and reliable way for TB+ and TB-?	8) Was appropriate statistical analysis used?
	9) Was follow up complete, and if not, were the reasons to loss to follow up described and explored?	9) Was the DM period of interest long enough to be meaningful?	
	10) Were strategies to address incomplete follow up utilized?	10) Was appropriate statistical analysis used?	
	11) Was appropriate statistical analysis used?		
Total score	**11**	10	8
**Interpretation**	• 9-11: Low risk of bias<br>• 5-8: Moderate risk of bias<br>• 0-4: High risk of bias	• 8-10: Low risk of bias<br>• 5-7: Moderate risk of bias<br>• 0-4: High risk of bias	• 7-8: Low risk of bias<br>• 4-6: Moderate risk of bias<br>• 0-3: High risk of bias
