# Supplementary material for: Diabetes mellitus and tuberculosis, a systematic review and meta-analysis with sensitivity analysis for studies comparable for confounders
Source: PLoS One. 2021 Dec 10;16(12):e0261246. doi: 10.1371/journal.pone.0261246 (PMC8664214; doi:10.1371/journal.pone.0261246)
Supplement: S4 Table — (PDF) [file pone.0261246.s007.pdf]

S4 Table. Main reasons of exclusion of eligible studies

N°	Author, Year	Title	Reason of exclusion
1	Abdelbary, 2016	Tuberculosis-diabetes epidemiology in the border and non-border regions of Tamaulipas, Mexico.	Incorrect study design
2	Abedi, 2019	The impact of diabetics and smoking on gender differences of smear positive pulmonary tuberculosis incidence.	Incorrect study design, No data on TB-
3	Abera, 2018	Pulmonary Tuberculosis and Associated Factors Among Diabetic Patients Attending Hawassa Adare Hospital, Southern Ethiopia.	Incorrect study design
4	Abou-Khatwa, 1963	COMPLICATIONS OF DIABETES MELLITUS (STUDY OF 2012 CASES).	No abstract and full text available
5	Abreu, 2020	Tuberculosis and diabetes: association with sociodemographic characteristics and diagnosis and treatment of tuberculosis. Brazil, 2007-2011.	Incorrect study design
6	Abreu, 2017	Tuberculosis and diabetes: probabilistic linkage of databases to study the association between both diseases.	Incorrect study design
7	Abu-Ashour, 2018	Diabetes and the occurrence of infection in primary care: a matched cohort study.	No outcome data
8	Achanta, 2013	Screening tuberculosis patients for diabetes in a tribal area in South India.	Incorrect study design
9	Achhra, 2014	Relationship between hyperglycemia and the risk of tuberculosis in Asian HIV-positive individuals in the antiretroviral therapy era: cohort study.	No DM - group, No data on TB-
10	Agarwal, 2016	The association between diabetes and tuberculosis may be the next challenge for global tuberculosis control worldwide.	Incorrect study population
11	Ahmed, 2017	Association between pulmonary tuberculosis and Type 2 diabetes in Sudanese patients.	No data on TB-
12	Airaghi, 2006	Negative association between occurrence of type 1 diabetes and tuberculosis incidence at population level.	No DM + group, No DM - group
13	Alavi, 2014	Comparison of epidemiological, clinical, laboratory and radiological features of hospitalized diabetic and non-diabetic patients with pulmonary tuberculosis at razi hospital in ahvaz.	Incorrect study design
14	Alavi, 2012	Pulmonary tuberculosis and diabetes mellitus: Co-existence of both diseases in patients admitted in a teaching hospital in the southwest of Iran.	Incorrect study population
15	Alimpic, 1970	Diabetes mellitus and pulmonary tuberculosis.	No abstract and full text available
16	Alvarez, 2014	Taima (stop) TB: the impact of a multifaceted TB awareness and door-to-door campaign in residential areas of high risk for TB in Iqaluit, Nunavut.	No data on TB-
17	Araujo, 2013	L-methionine placental uptake: characterization and modulation in gestational diabetes mellitus.	Incorrect study design
18	Arnedo-pena, 2015	Vitamin D status and incidence of tuberculosis infection conversion in contacts of pulmonary tuberculosis patients: a prospective cohort study.	Incorrect study design
19	Asanov, 1997	Surgical treatment of pulmonary tuberculosis in patients with diabetes mellitus.	No abstract and full text available
20	Asante-poku, 2019	TB-diabetes co-morbidity in Ghana: The importance of Mycobacterium africanum infection.	Incorrect study design
21	Asberg, 2006	Calcineurin inhibitor avoidance with daclizumab, mycophenolate mofetil, and prednisolone in DR-matched de novo kidney transplant recipients.	Incorrect study population
22	Baghaei, 2009	Risk Factors Associated with Multidrug-Resistant Tuberculosis.	No data on outcome
23	Balde, 2006	Associated tuberculosis and diabetes in Conakry, Guinea: Prevalence and clinical characteristics.	Incorrect study design

24	Bashar, 2001	Increased incidence of multidrug-resistant tuberculosis in diabetic patients on the Bellevue Chest Service, 1987 to 1997.	Incorrect study population
25	Bashar, 2001	Increased incidence of multidrug-resistant tuberculosis in diabetic patients on the Bellevue Chest Service, 1987 to 1997.	Duplicate study
26	Bennett, 2014	Prevalence and treatment of latent tuberculosis infection among newly arrived refugees in San Diego County, January 2010-October 2012.	Incorrect study design
27	Benoît, 2017	Association of Diabetes and Tuberculosis Disease among US-Bound Adult Refugees, 2009-2014.	No data on TB-
28	Boillat-blanco, 2016	Association between tuberculosis, diabetes and 25 hydroxyvitamin D in Tanzania: A longitudinal case control study.	Duplicate study
29	Bridon, 2015	Diabetes: A Contributor to Tuberculosis in Tropical Australia.	No data on TB-
30	Bronz, 2019	Association between diabetes mellitus and mortality among patients with tuberculosis.	Comment on an article
31	Byberg, 2012	Diabetes is a risk factor for tuberculosis in the Inuit population of Greenland.	Editorial
32	Carriera , 2012	Impact of diabetes on the presenting features of tuberculosis in hospitalized patients.	Not write in english or in french
33	Carrion-Torres, 2015	Characteristics of the diagnosis and treatment of pulmonary tuberculosis in patients with and without diabetes mellitus type 2.	Incorrect study population
34	Cegielki, 2012	Nutritional risk factors for tuberculosis among adults in the United States, 1971-1992.	No data on DM
35	Chang, 2011	Effect of type 2 diabetes mellitus on the clinical severity and treatment outcome in patients with pulmonary tuberculosis: a potential role in the emergence of multidrug-resistance.	Incorrect study population
36	Chan-yeung, 2006	Prevalence and determinants of positive tuberculin reactions of residents in old age homes in Hong Kong.	Not full text
37	Goldhaber- Fiebert, 2011	Diabetes mellitus and tuberculosis in countries with high tuberculosis burdens: individual risks and social determinants.	Duplicate study
38	Chen , 2013	Pulmonary tuberculosis incidence and risk factors in rural areas of China: a cohort study.	Incorrect study design
39	Chukanova, 2000	Epidemiological and immunogenetic analysis of tuberculosis and diabetes mellitus association.	Not in french or english
40	Coker, 2006	Risk factors for pulmonary tuberculosis in Russia: case-control study.	Not possible to extract data from TB & DM
41	Defang, 2014	Screening for tuberculosis and LTBI in diabetes patients, Pohnpei, Federated States of Micronesia.	Incorrect study population
42	Delgado- Sanchez, 2015	Association of Pulmonary Tuberculosis and Diabetes in Mexico: Analysis of the National Tuberculosis Registry 2000-2012.	Incorrect study population
43	Demlow, 2015	Increased risk of tuberculosis among foreign-born persons with diabetes in California, 2010-2012.	Incorrect study population
44	Deshmukh, 2020	Assessment of the Xpert assay among adult pulmonary tuberculosis suspects with and without diabetes mellitus.	Incorrect study design
45	Destiany, 2020	Risk factor of pulmonary tuberculosis among people with diabetes mellitus in Makassar.	Incorrect study design
46	Dobler, 2012	Risk of tuberculosis among people with diabetes mellitus: an Australian nationwide cohort study.	Incorrect study population
47	Du, 2017	Clinical significance of serum CA-125, CA19-9 and CEA in pulmonary tuberculosis with and without type 2 diabetes.	No abstract and full text available
48	Dyck, 2007	The relationship between diabetes and tuberculosis in Saskatchewan - Comparison of registered Indians and other Saskatchewan people.	Incorrect study design
49	Fisher- Hoch, 2008	Type 2 diabetes and multidrug-resistant tuberculosis.	Incorrect study population
50	Fossati, 1965	ON THE ASSOCIATION OF PULMONARY TUBERCULOSIS AND DIABETES MELLITUS. RESULTS	Not found

		OBTAINED ADMINISTERING TO THESE PATIENTS AN ORAL ANTIDIABETIC.	
51	Fossati, 1969	[Associated diabetes and pulmonary tuberculosis: occurrence, development, mortality and causes of death; statistical study of Arab-Libyan patients in Cyrene and covering the 10 past years].	No abstract and full text available
52	Gedfew, 2020	Incidence and Predictors of Tuberculosis among Adult Diabetic Patients, Debre Markos Referral Hospital, Northwest Ethiopia, 2018: A Retrospective Cohort Study.	No DM - group
53	Goldhalber- Fiebert, 2011	Diabetes mellitus and tuberculosis in countries with high tuberculosis burdens: individual risks and social determinants.	Incorrect study population
54	Gomez-Gomez, 2015	Diabetes and Other Risk Factors for Multi-drug Resistant Tuberculosis in a Mexican Population with Pulmonary Tuberculosis: Case Control Study.	Incorrect study population
55	Greuel, 1958	Active pulmonary tuberculosis & diabetes mellitus.	No abstract and full text available
56	Hafez, 2013	Detection of extensively drug resistant pulmonary tuberculosis.	Incorrect study population
57	HARRIES, 2019	Having diabetes and being underweight in Asia: a potent risk factor for tuberculosis.	Editorial
58	Hsu, 2012	Diabetes is associated with drug-resistant tuberculosis in Eastern Taiwan Short communication.	Incorrect study population
59	Hu, 2013	Increased risk of tuberculosis in patients with end-stage renal disease: a population-based cohort study in Taiwan, a country of high incidence of end-stage renal disease.	Incorrect study population
60	Jackson, 2013	S57 Diabetes and latent tuberculosis infection: nested case-control study within the PREDICT cohort.	not possible to extract data on outcome
61	Jacob, 2017	Clincal interpretation of the anti-tuberculosis drug concentrations in diabetic and non-diabetic tuberculosis patients.	Incorrect study population
62	Jinhong, 2005	Risk Factors for Primary Multidrug Resistant Tuberculosis.	Incorrect study population
63	Jitmuang, 2015	PREVALENCE AND FACTORS ASSOCIATED WITH MULTIDRUG-RESISTANT TUBERCULOSIS AT SIRIRAJ HOSPITAL, BANGKOK, THAILAND.	Incorrect study population
64	John, 2001	Risk factors for post-transplant tuberculosis.	Incorrect study population
65	Kamper-Jørgensen, 2015	Diabetes-related tuberculosis in Denmark: effect of ethnicity, diabetes duration and year of diagnosis.	Incorrect study population
66	kennedy, 1933	ACTIVE PULMONARY TUBERCULOSIS AND DIABETES MELLITUS.	Review
67	Khalil, 2016	Study of risk factors for pulmonary tuberculosis among diabetes mellitus patients.	No DM - group
68	Kim, 2019	Association between Tuberculosis, Statin Use, and Diabetes: A Propensity Score-Matched Analysis.	No data on TB-
69	Ku, 2013	Incidence of and risk factors for active tuberculosis in human immunodeficiency virus-infected patients in South Korea.	Incorrect study population
70	Kurgan, 1966	Diabetes insipidus in the course of miliary, pulmonary tuberculosis.	No abstract and full text available
71	Lawson, 2017	Tuberculosis and diabetes in Nigerian patients with and without HIV.	not possible to extract data on outcome
72	Lebon, 1965	[Diabetes mellitus and tuberculosis in Algerian Moslems].	No abstract and full text available
73	Lee, 2013	Risk factors for pulmonary tuberculosis in patients with chronic obstructive airway disease in Taiwan: a nationwide cohort study.	Incorrect study population
74	Lee, 2010	High prevalence of latent tuberculosis infection in dialysis patients using the interferon-gamma release assay and tuberculin skin test.	Incorrect study design
75	Levin, 1959	Diabetes insipidus associated with tuberculous meningitis.	Case report

76	Li, 2015	Factors associated with primary transmission of multidrug-resistant tuberculosis compared with healthy controls in Henan Province, China.	Impossible to extract data on exposure
77	Li, 2020	Incidence of pulmonary tuberculosis in Chinese adults with type 2 diabetes: a retrospective cohort study in Shanghai.	No DM - group
78	Lin, 2018	Association of Obesity, Diabetes, and Risk of Tuberculosis: Two Population-Based Cohorts.	No data on TB-
79	Maâlej, 2009	[Pulmonary tuberculosis and diabetes. A retrospective study of 60 patients in Tunisia].	Incorrect study population
80	Magee, 2013	Clinical characteristics, drug resistance, and treatment outcomes among tuberculosis patients with diabetes in Peru.	Incorrect study population
81	Magee, 2015	Diabetes mellitus is associated with cavities, smear grade, and multidrug-resistant tuberculosis in Georgia.	No data on TB-
82	Marks, 2011	Diabetes and tuberculosis, US National Health Interview Survey, 2000-2005.	Incorrect study design
83	Martinez-Pulgarin, 2014	Tuberculosis and diabetes comorbidity in a hospital of Colombia.	Comment on an article
84	Marton, 1969	Clinical-pathological studies of lung resected tuberculotic diabetics. I. Manifestation and clinical course of the diabetes-tuberculosis and their consequences.	No abstract and full text available
85	Marton, 1969	Clinical pathological studies of lung resected tuberculotic diabetics. II. Relation between the factors of diabetes and the clinical course of tuberculosis.	No abstract and full text available
86	Marzi, 1970	Clinico-therapeutic observations on the association of diabetes and tuberculosis in adult, presenile and senile subjects.	No abstract and full text available
87	Marzi, 1968	Relationship between diabetes and tuberculosis in the aged.	No abstract and full text available
88	Masztalerz, 1990	The role of diabetes as a factor for increased risk of infection with tuberculosis.	Incorrect study design
89	McAllister, 2020	High tuberculosis incidence among people living with diabetes in Indonesia.	Incorrect study design
90	Mendoza-Aguilar, 2012	Functional state analysis of phagocytic cells of patients with type 2 diabetes and pulmonary tuberculosis.	Incorrect study design
91	Mi, 2014	Is resistance to anti-tuberculosis drugs associated with type 2 diabetes mellitus? A register review in Beijing, China.	Incorrect study design
92	Min, 2005	Risk Factors for Primary Multidrug Resistant Tuberculosis.	No abstract and full text available
93	Montenero, 1950	Interocurrence between diabetes and tuberculosis.	No abstract and full text available
94	Morán-Mendoza, 2010	Risk factors for developing tuberculosis: a 12-year follow-up of contacts of tuberculosis cases.	Incorrect study population
95	Moualeu, 2012	Analysis of The Impact of Diabetes on The Dynamical Transmission of Tuberculosis.	Incorrect study design
96	Mu, 2019	Screening for the Interaction of Diabetes Mellitus and Other Factors in Tuberculosis.	Incorrect study population
97	Muhammad, 2007	Features of pulmonary tuberculosis in patients with diabetes mellitus: a comparative study.	Incorrect study population
98	Mukhtar, 2016	Cohort profile: The diabetes-tuberculosis treatment outcome (DITTO) study in Pakistan.	Incorrect study population
99	Nagar, 2018	A study on prevalence of diabetes and associated risk factors among diagnosed tuberculosis patients registered under Revised National Tuberculosis Control Programme in Bhopal District.	Incorrect study population
100	Nakamura, 2014	Impact of underlying diabetes and presence of lung cavities on treatment outcomes in patients with pulmonary tuberculosis.	Incorrect study population
101	Nanda, 1968	Association of diabetes mellitus with pulmonary tuberculosis.	No abstract and full text available
102	Nasa, 2014	Screening adult tuberculosis patients for diabetes mellitus in Ebeye, Republic of the Marshall Islands.	Incorrect study design

103	No author listed, 1957	TUBERCULOSIS and diabetes.	No abstract and full text available
104	No author listed, 1971	Pulmonary tuberculosis, sepsis, hematuria, uremia, diabetes and death.	Case report
105	No author listed, 1974	Tuberculosis and diabetes mellitus.	No abstract and full text available
106	No author listed, 1979	Infection and diabetes mellitus.	No abstract and full text available
107	No author listed, 2010	Evaluating the epidemiology of the tuberculosis and diabetes epidemics.	No abstract and full text available
108	No author listed, 2013	Screening of patients with diabetes mellitus for tuberculosis in India.	Incorrect study population
109	No author listed, 2013	Screening of patients with tuberculosis for diabetes mellitus in India.	Duplicate study
110	Ocal, 2009	Mild and severe forms of tuberculosis in diabetic and non-diabetic patients.	No data on TB-
111	Olmos, 1989	Tuberculosis and diabetes mellitus: a longitudinal-retrospective study in a teaching hospital.	No DM - group
112	Ou, 2012	Impact of pulmonary and extrapulmonary tuberculosis infection in kidney transplantation: a nationwide population-based study in Taiwan.	Incorrect study population
113	Pablo-Villamor Maria, 2014	Screening for diabetes mellitus in patients diagnosed with pulmonary tuberculosis.	No data on TB-
114	Pagliara, 1966	Aspects of the association of diabetes and tuberculosis observed in the dispensary.	No abstract and full text available
115	Pearson, 2019	Tuberculosis and diabetes: bidirectional association in a UK primary care data set.	No DM - group, No data on TB-
116	Perez-Navarro, 2017	The effect size of type 2 diabetes mellitus on tuberculosis drug resistance and adverse treatment outcomes.	Incorrect study population
117	Pérez-Navarro, 2015	Type 2 diabetes mellitus and its influence in the development of multidrug resistance tuberculosis in patients from southeastern Mexico.	Incorrect study population
118	Ponce-De-Leon, 2004	Tuberculosis and diabetes in southern Mexico.	Incorrect study population
119	Qiu, 2017	Incident rate and risk factors for tuberculosis among patients with type 2 diabetes: retrospective cohort study in Shanghai, China.	No DM - group
120	Rajan, 2017	Diabetes increases the risk of recent-transmission tuberculosis in household contacts in Sao Paulo, Brazil.	Incorrect study population
121	Rajan, 2017	Diabetes increases the risk of recent-transmission tuberculosis in household contacts in São Paulo, Brazil.	Duplicate study
122	Rifat, 2014	Development of multidrug resistant tuberculosis in Bangladesh: a case-control study on risk factors.	Incorrect study population
123	Rosenman, 1996	Occupational risk factors for developing tuberculosis.	Incorrect study population
124	Ruesen, 2020	Diabetes is associated with genotypically drug-resistant tuberculosis.	Incorrect study population
125	Rungruanghiranya, 2008	Tuberculosis in Thai renal transplant recipients: a 15-year experience.	Incorrect study population
126	Russom, 2019	Risk Factors of Gout in MDR-TB Patients in Eritrea: A Case-Control Study.	Incorrect study population
127	Saktiawati, 2018	Influence of Diabetes Mellitus on the Development of Multi Drug Resistant-Tuberculosis in Yogyakarta.	Incorrect study population
128	Salindri, 2016	Diabetes Reduces the Rate of Sputum Culture Conversion in Patients With Newly Diagnosed Multidrug-Resistant Tuberculosis.	Incorrect study population
129	Sembiah, 2020	Diabetes in tuberculosis patients: An emerging public health concern and the determinants and impact on treatment outcome.	No data on TB-

130	Sfirleaza, 1976	Inter-relations between pulmonary tuberculosis and diabetes mellitus.	No data on TB-
131	Shanmuganathan, 2015	Clinical manifestation and risk factors of tuberculosis infection in Malaysia: case study of a community clinic.	Incorrect study population
132	Shetty, 2006	An epidemiological evaluation of risk factors for tuberculosis in South India: a matched case control study.	No data on DM
133	India, 2020	Co-existence of diabetes and TB among adults in India: a study based on National Family Health Survey data.	Not possible to collect data on exposure
134	Singh, 2016	Association of tuberculosis and diabetes Mellitus: an analysis of 1000 consecutively admitted cases in a tertiary care hospital of North India.	Incorrect study population
135	Singla, 2003	Does diabetes predispose to the development of multidrug-resistant tuberculosis?	Comment on an article
136	Singla, 2006	Influence of diabetes on manifestations and treatment outcome of pulmonary TB patients.	No data on TB-
137	Skodric-Trifunovic, 2004	Risk factors for developing tuberculosis.	Review
138	Skodric-Trifunovic, 2004	Analysis of patients with tuberculosis and diabetes mellitus at the Institute of Pulmonary Diseases and Tuberculosis of the Clinical Center of Serbia (2000-2002).	Article not in English or French
139	Soh, 2019	Diabetes and body mass index in relation to risk of active tuberculosis: a prospective population-based cohort.	No data on TB
140	Stosic, 2018	Risk factors for multidrug-resistant tuberculosis among tuberculosis patients in Serbia: a case-control study.	No data on TB-
141	Suárez-García, 2009	Risk factors for multidrug-resistant tuberculosis in a tuberculosis unit in Madrid, Spain.	No data on TB-
142	Tanrikulu, 2008	Risk factors for drug resistant tuberculosis in southeast Turkey.	No data on TB-
143	Tarasov, 1975	Association of diabetes mellitus and pulmonary tuberculosis (based on sanatorium data).	Article not in English or in French
144	Teng, 2019	Increased risk of tuberculosis in oral cancer patients in an endemic area: a nationwide population-based study.	No data on DM
145	Tessier, 1962	[Association of a sarcoidosis with diabetes insipidus and pulmonary tuberculosis (observation--comments)].	No abstract and full text available
146	Viney, 2019	Tuberculosis and diabetes mellitus: A dose-response relationship between the odds of tuberculosis and HbA1c.	Duplicate study
147	Viswanathan, 2014	Effect of type II diabetes mellitus on treatment outcomes of tuberculosis.	Incorrect study population
148	Weng, 2009	Extrapulmonary tuberculosis: a study comparing diabetic and nondiabetic patients.	Incorrect study population
149	Wu, 2011	Aerodigestive tract, lung and haematological cancers are risk factors for tuberculosis: an 8-year population-based study.	Incorrect study population
150	Yamamura, 1964	ASSOCIATION OF PULMONARY TUBERCULOSIS AND DIABETES MELLITUS.	Article not in English or in French
151	Yang, 2018	Regional differences in the incidence of tuberculosis among patients with newly diagnosed diabetes mellitus.	No DM - group
152	Young, 2012	Increased risk of tuberculosis disease in people with diabetes mellitus: record-linkage study in a UK population.	Incorrect study population
153	Zhang, 2009	Tuberculosis complicated by diabetes mellitus at shanghai pulmonary hospital, china.	Incorrect study population
154	Zhu, 2006	Risk factors for the development of pulmonary tuberculosis among type 2 diabetes mellitus patients.	Incorrect study population
