# Supplementary material for: Diabetes mellitus and tuberculosis, a systematic review and meta-analysis with sensitivity analysis for studies comparable for confounders
Source: PLoS One. 2021 Dec 10;16(12):e0261246. doi: 10.1371/journal.pone.0261246 (PMC8664214; doi:10.1371/journal.pone.0261246)
Supplement: S5 Table — (PDF) [file pone.0261246.s008.pdf]

S5 Table. Risk of bias assessment

Author, Year	Study Design	Were the DM+ and DM- groups similar and recruited from the same population?	Were the DM measured similarly to assign people to both DM+ and DM- groups?	Was the DM measured in a valid and reliable way?	Were confounding factors identified?	Were strategies to deal with confounding factors stated?	Were the DM+ and DM- free of the TB at the start of the study (or at the moment of DM)?	Were the TB measured in a valid and reliable way?	Was the follow up time reported and sufficient to be long enough for TB to occur?	Was follow up complete, and if not, were the reasons to loss to follow up described and explored?	Were strategies to address incomplete follow up utilized?	Was appropriate statistical analysis used?	Were the TB+ and TB- groups comparable other than presence of TB in cases or absence of TB in controls?	Were TB+ and TB- matched appropriately?	Were the same criteria used for identification of TB+ and TB-?	Was DM measured in a standard, valid and reliable way?	Was DM measured in the same way for TB+ and TB-?	Were confounding factors identified?	Were strategies to deal with confounding factors stated?	Were TB assessed in a standard, valid and reliable way for TB+ and TB-?	Was the DM period of interest long enough to be meaningful?	Was appropriate statistical analysis used?	Were the criteria for inclusion in the sample clearly defined?	Were the study subjects and the setting described in detail?	Was the DM measured in a valid and reliable way?	Were objective, standard criteria used for measurement of the condition?	Were confounding factors identified?	Were strategies to deal with confounding factors stated?	Were the TB measured in a valid and reliable way?	Was appropriate statistical analysis used?	Risk of biais
Alisiahbana, 2006	Case control												Yes	Yes	Yes	Yes	Yes	Yes	Yes	Yes	Yes	Yes									Low risk of bias
Boillat-blanco, 2016	Case control												Yes	Yes	No	Yes	Yes	Yes	Yes	Yes	No	Yes									Low risk of bias
Brassard, 2006	Case control												Yes	Yes	Yes	Yes	Yes	Yes	Yes	Yes	Yes	Yes									Low risk of bias
Buskin, 1994	Case control												No	No	Yes	Unclear	Unclear	Yes	Yes	No	Unclear	Yes									High risk of bias
Chung, 2014	Case control												Yes	Yes	Yes	Yes	Yes	Yes	Yes	Yes	Yes	Yes									Low risk of bias
Corris, 2012	Case control												No	No	Yes	Yes	No	Yes	Yes	Yes	Unclear	Yes									Moderate risk of bias
Davis, 2017	Case control												Yes	No	Yes	Yes	Yes	Yes	Yes	Yes	Unclear	Yes									Low risk of bias
Faurholt-Jepsen, 2011	Case control												Yes	Yes	Yes	No	Yes	Yes	Yes	Yes	Unclear	Yes									Low risk of bias
Haraldsdottir, 2015	Case control												No	No	No	Yes	Yes	Yes	Yes	Yes	Yes	Yes									Moderate risk of bias
Hossain, 2014	Case control												No	No	No	Yes	Yes	No	No	Yes	Yes	No									High risk of bias
Jick, 2006	Case control												Yes	Yes	Yes	Yes	Yes	Yes	Yes	Yes	Yes	Yes									Low risk of bias
Jurcev-Savicevic, 2013	Case control												Yes	Yes	Yes	No	Unclear	Yes	Yes	Yes	Yes	Yes									Low risk of bias
Lai, 2014	Case control												Yes	Yes	Yes	Yes	Yes	Yes	Yes	Yes	Yes	Yes									Low risk of bias
Lee, 2013	Case control												No	No	Yes	Yes	Yes	Yes	Yes	Yes	Yes	Yes									Low risk of bias
Leegaard, 2011	Case control												Yes	Yes	Yes	Yes	Yes	Yes	Yes	Yes	Yes	Yes									Low risk of bias
Mori, 1992	Case control												No	No	Yes	Yes	Yes	Yes	Yes	Unclear	Yes	Yes									Moderate risk of bias
Ndishimye, 2017	Case control												Yes	Yes	No	No	No	No	No	Unclear	Unclear	Yes									High risk of bias
Pablos-Méndez, 1997	Case control												No	No	Yes	Yes	Yes	No	No	Yes	Unclear	No									High risk of bias
Pereira, 2016	Case control												Yes	Yes	Yes	Yes	Yes	Yes	No	Yes	Unclear	Yes									Low risk of bias
Pérez, 2006	Case control												No	No	Yes	Yes	Yes	No	No	Unclear	Unclear	Yes									High risk of bias
Shimouchi, 2020	Case control												Yes	No	Yes	Yes	Yes	Yes	Yes	Yes	Unclear	Yes									Low risk of bias
Viney, 2015	Case control												No	No	Yes	Yes	Yes	Yes	Yes	Yes	Unclear	Yes									Moderate risk of bias
Zhussupov, 2016	Case control												Yes	Yes	No	No	No	Yes	Yes	Yes	Unclear	Yes									Moderate risk of bias
Golub, 2019	Cohort	Yes	Yes	Yes	Yes	Yes	Yes	Yes	Yes	Yes	Not applicable	Yes																			Low risk of bias
Kim, 1995	Cohort	Unclear	Yes	Yes	No	Not applicable	Unclear	Yes	Yes	Unclear	Unclear	Yes																			Moderate risk of bias
Kuo, 2013	Cohort	Yes	Yes	Yes	Yes	Yes	Yes	Yes	Yes	Yes	Yes	Yes																			Low risk of bias
Lee, 2014	Cohort	Yes	Yes	Yes	Yes	Yes	Yes	Yes	Yes	Yes	Not applicable	Yes																			Low risk of bias
Lee, 2016	Cohort	Yes	Yes	Yes	Yes	Yes	Yes	Yes	Yes	Unclear	Unclear	Yes																			Low risk of bias
Leung, 2008	Cohort	Yes	Yes	Yes	Yes	Yes	Yes	Yes	Yes	Yes	Not applicable	Yes																			Low risk of bias
Lin, 2017	Cohort	Yes	Yes	Yes	Yes	Yes	Yes	Yes	Yes	Yes	Not applicable	Yes																			Low risk of bias
Lin, 2019	Cohort	No	Yes	Yes	Yes	No	Yes	No	Yes	Unclear	No	Yes																			Moderate risk of bias
Pealing, 2015	Cohort	Yes	Yes	Yes	Yes	Yes	Yes	Yes	Yes	Yes	Not applicable	Yes																			Low risk of bias
Shen, 2014	Cohort	Yes	Yes	Yes	No	Not applicable	Yes	Yes	Yes	Unclear	Unclear	Yes																			Moderate risk of bias
Baker, 2012	Cross sectional																						Yes	Yes	Yes	Yes	Yes	Yes	Yes	Yes	Low risk of bias
Barron, 2018 - Diabetes	Cross sectional																						Yes	Yes	Yes	Yes	Yes	Yes	Yes	Yes	Low risk of bias
Barron, 2018 - Prediabetes	Cross sectional																						Yes	Yes	Yes	Yes	Yes	Yes	Yes	Yes	Low risk of bias
Chen, 2006	Cross sectional																						Yes	No	Unclear	Yes	Yes	No	Yes	No	Moderate risk of bias
Faurholt-Jensen, 2014	Cross sectional																						No	No	Yes	Yes	Yes	Yes	Yes	Yes	Moderate risk of bias
Hensel, 2016	Cross sectional																						Yes	Yes	Yes	Yes	Yes	Yes	Yes	Yes	Low risk of bias
Khawcharoenporn, 2015	Cross sectional																						Yes	Yes	Unclear	Yes	Yes	Yes	Yes	Yes	Low risk of bias
Kubiak, 2019	Cross sectional																						Yes	Yes	Yes	Yes	Yes	Yes	Yes	Yes	Low risk of bias
Shu, 2012	Cross sectional																						Yes	Yes	Unclear	Yes	Yes	Yes	Yes	Yes	Low risk of bias
Suwanpimolkul, 2014 - Active TB	Cross sectional																						Yes	Yes	Yes	Yes	Yes	Yes	Yes	Yes	Low risk of bias
Suwanpimolkul, 2014 - LTBI	Cross sectional																						Yes	Yes	Yes	Yes	Yes	Yes	Yes	Yes	Low risk of bias
Swarna Nantha, 2017	Cross sectional																						Yes	Yes	Yes	Yes	Yes	Yes	Yes	Yes	Low risk of bias
Ting, 2014	Cross sectional																						Yes	Yes	Unclear	Yes	Yes	Yes	Yes	Yes	Low risk of bias
Wang, 2013	Cross sectional																						Yes	Yes	Yes	Yes	Yes	Yes	Yes	Yes	Low risk of bias
Wardhani, 2019	Cross sectional																						Yes	Yes	No	No	Yes	No	No	No	High risk of bias
Wu, 2007	Cross sectional																						Yes	Yes	Unclear	Yes	Yes	No	Yes	No	Moderate risk of bias
