# Supplementary material for: Diabetes mellitus and tuberculosis, a systematic review and meta-analysis with sensitivity analysis for studies comparable for confounders
Source: PLoS One. 2021 Dec 10;16(12):e0261246. doi: 10.1371/journal.pone.0261246 (PMC8664214; doi:10.1371/journal.pone.0261246)
Supplement: S6 Table — (PDF) [file pone.0261246.s009.pdf]

S6 Table. P-value of Khi-2 and Fisher exact tests for qualitative confounding factors

		Data extracted from included studies				Results from this study		
Author, Year	Qualitative confounding factors	Total number of reference group	Number of reference group with confounding factors	Total number of control group	Number of control group with confounding factors	P-value Khi-2 test	P-value Fisher exact test	Status
Lai, 2014	Adenotonsillectomy	11366	7	45464	31	0,968	1,000	Symmetric
Boillat-blanco, 2016	Anemia	530	425	491	172	0,000	0,000	Asymmetric
Shu, 2012	Anemia	91	64	316	240	0,342	0,277	Symmetric
Lai, 2014	Appendectomy	11366	123	45464	320	0,000	0,000	Asymmetric
Lin, 2017	Asbestosis	22256	15	89024	69	0,723	0,684	Symmetric
Swarna Nantha, 2017	Atrial fibrillation	404	2	359	0	0,532	0,501	Symmetric
Lee, 2013	Autoimmune disorders	49903	104	49903	133	0,069	0,068	Symmetric
Shu, 2012	Autoimmune disorders	91	2	316	9	1,000	1,000	Symmetric
Ting, 2014	Autoimmune disorders	296	34	722	106	0,214	0,194	Symmetric
Swarna Nantha, 2017	Benign prostatic hyperplasia	404	0	359	7	0,015	0,005	Asymmetric
Lee, 2016	Bet nut use	11260	292	110782	2553	0,057	0,057	Symmetric
Swarna Nantha, 2017	Bronchial asthma	404	4	359	6	0,612	0,529	Symmetric
Wu, 2007	Bronchiectasis	264	5	438	8	1,000	1,000	Symmetric
Baker, 2012	Cardiovascular disease	1652	356	16063	881	0,000	0,000	Asymmetric
Boillat-blanco, 2016	Cardiovascular disease	530	44	491	74	0,001	0,001	Asymmetric
Chung, 2014	Cardiovascular disease	10168	1692	40672	5583	0,000	0,000	Asymmetric
Leung, 2008	Cardiovascular disease	6444	1282	35672	4780	0,000	0,000	Asymmetric
Swarna Nantha, 2017	Cardiovascular disease	404	0	359	5	0,054	0,023	Asymmetric
Jurcev-Savicevic, 2013	Central sewage system	300	198	300	185	0,308	0,308	Symmetric
Jurcev-Savicevic, 2013	Central water supply	300	265	300	245	0,030	0,029	Asymmetric
Golub, 2019	Chronic kidney disease	52820	30	766231	418	0,907	0,773	Symmetric
Jurcev-Savicevic, 2013	Chronic kidney disease	300	1	300	2	1,000	1,000	Symmetric
Lai, 2014	Chronic kidney disease	11366	535	45464	1054	0,000	0,000	Asymmetric
Lee, 2013	Chronic kidney disease	49903	160	49903	104	0,001	0,001	Asymmetric
Lee, 2014	Chronic kidney disease	300	6	300	4	0,750	0,752	Symmetric
Lee, 2016	Chronic kidney disease	11260	40	110782	81	0,000	0,000	Asymmetric
Lin, 2017	Chronic kidney disease	22256	657	89024	1137	0,000	0,000	Asymmetric
Lin, 2019	Chronic kidney disease	2948	948	453	57	0,000	0,000	Asymmetric
Mori, 1992	Chronic kidney disease	46	6	46	0	0,035	0,026	Asymmetric
Pablos-Méndez, 1997	Chronic kidney disease	5290	154	37366	121	0,000	0,000	Asymmetric
Pérez, 2006	Chronic kidney disease	4915	75	70808	308	0,000	0,000	Asymmetric
Shen, 2014	Chronic kidney disease	5195	380	20780	249	0,000	0,000	Asymmetric
Swarna Nantha, 2017	Chronic kidney disease	404	1	359	1	1,000	1,000	Symmetric
Ting, 2014	Chronic kidney disease	296	7	722	15	0,961	0,813	Symmetric

Zhussupov, 2016	Chronic kidney disease	110	7	214	27	0,122	0,088	Symmetric
Lai, 2014	Chronic liver disease	11366	1901	45464	5345	0,000	0,000	Asymmetric
Lin, 2017	Chronic liver disease	22256	1128	89024	1348	0,000	0,000	Asymmetric
Shen, 2014	Chronic liver disease	5195	491	20780	1306	0,000	0,000	Asymmetric
Swarna Nantha, 2017	Chronic liver disease	404	0	359	1	0,953	0,471	Symmetric
Chung, 2014	Chronic obstructive pulmonary disease	10168	5463	40672	11600	0,000	0,000	Asymmetric
Lai, 2014	Chronic obstructive pulmonary disease	11366	4409	45464	7223	0,000	0,000	Asymmetric
Lee, 2013	Chronic obstructive pulmonary disease	49903	3082	49903	2099	0,000	0,000	Asymmetric
Leung, 2008	Chronic obstructive pulmonary disease	6444	328	35672	2104	0,011	0,011	Asymmetric
Lin, 2017	Chronic obstructive pulmonary disease	22256	4534	89024	12653	0,000	0,000	Asymmetric
Swarna Nantha, 2017	Chronic obstructive pulmonary disease	404	2	359	1	1,000	1,000	Symmetric
Ting, 2014	Chronic obstructive pulmonary disease	296	18	722	15	0,002	0,003	Asymmetric
Jurcev-Savicevic, 2013	Co-morbidity	300	144	300	141	0,870	0,870	Symmetric
Shen, 2014	Co-morbidity	5195	1714	20780	4438	0,000	0,000	Asymmetric
Zhussupov, 2016	Currently rent home	110	20	214	24	0,118	0,089	Symmetric
Baker, 2012	Drinker	1652	142	16063	1364	0,922	0,889	Symmetric
Boillat-blanco, 2016	Drinker	530	62	491	27	0,001	0,001	Asymmetric
Buskin, 1994	Drinker	151	39	545	68	0,000	0,000	Asymmetric
Davis, 2017	Drinker	562	279	1038	400	0,000	0,000	Asymmetric
Haraldsdottir, 2015	Drinker	110	5	572	14	0,364	0,211	Symmetric
Hensel, 2016	Drinker	221	44	473	103	0,645	0,619	Symmetric
Jurcev-Savicevic, 2013	Drinker	300	130	300	140	0,460	0,460	Symmetric
Kubiak, 2019	Drinker	919	426	1113	70	0,000	0,000	Asymmetric
Lee, 2014	Drinker	300	78	300	42	0,000	0,000	Asymmetric
Lee, 2016	Drinker	11260	5514	110782	42002	0,000	0,000	Asymmetric
Leegaard, 2011	Drinker	2950	268	14274	216	0,000	0,000	Asymmetric
Leung, 2008	Drinker	6444	168	35672	1463	0,000	0,000	Asymmetric
Lin, 2017	Drinker	22256	297	89024	606	0,000	0,000	Asymmetric
Lin, 2017	Drinker	22256	525	89024	678	0,000	0,000	Asymmetric
Mori, 1992	Drinker	46	25	46	15	0,058	0,058	Symmetric
Ndishimye, 2017	Drinker	150	84	150	91	0,482	0,482	Symmetric
Pablos-Méndez, 1997	Drinker	5290	440	37366	172	0,000	0,000	Asymmetric
Pealing, 2015	Drinker	222731	18843	1218616	99444	0,000	0,000	Asymmetric
Pérez, 2006	Drinker	4915	227	70808	360	0,000	0,000	Asymmetric
Shimouchi, 2020	Drinker	192	88	190	59	0,004	0,003	Asymmetric
Wang, 2013	Drinker	6382	414	6675	753	0,000	0,000	Asymmetric
Davis, 2017	Drug user	562	5	1038	14	0,570	0,479	Symmetric
Jurcev-Savicevic, 2013	Drug user	300	15	300	7	0,128	0,127	Symmetric
Ndishimye, 2017	Drug user	150	7	150	10	0,617	0,619	Symmetric
Pablos-Méndez, 1997	Drug user	5290	183	37366	48	0,000	0,000	Asymmetric
Swarna Nantha, 2017	Dyslipidaemia	404	272	359	169	0,000	0,000	Asymmetric
Davis, 2017	Ever injected heroin	562	5	1038	5	0,512	0,335	Symmetric

Davis, 2017	Ever smoked heroin	562	1	1038	3	1,000	1,000	Symmetric
Davis, 2017	Ever used marijuana	562	26	1038	18	0,001	0,001	Asymmetric
Davis, 2017	Ever used opium	562	7	1038	4	0,095	0,059	Symmetric
Lee, 2014	Extra pulmonary lesion	300	51	300	48	0,826	0,826	Symmetric
Hossain, 2014	Family history of diabete mellitus	50	20	50	30	0,072	0,071	Symmetric
Ting, 2014	Fibro-calcified lesions on chest radiogram	296	86	722	128	0,000	0,000	Asymmetric
Jurcev-Savicevic, 2013	Food shortage	300	12	300	26	0,029	0,028	Asymmetric
Swarna Nantha, 2017	Gout	404	3	359	6	0,395	0,319	Symmetric
Zhussupov, 2016	Have children	110	70	214	191	0,000	0,000	Asymmetric
Lin, 2019	HDL, low	2948	1336	453	299	0,000	0,000	Asymmetric
Ting, 2014	Health care worker	296	15	296	27	0,078	0,077	Symmetric
Shu, 2012	Hemodialysis patients	91	67	316	218	0,471	0,437	Symmetric
Wu, 2007	Hemodialysis patients	264	3	438	7	0,864	0,750	Symmetric
Barron-HbA1c diabetes, 2018	Hepatitis B infection, HBsAb	293	41	4665	187	0,000	0,000	Asymmetric
Barron-HbA1c prediabetes, 2018	Hepatitis B infection, HBsAb	293	41	4665	187	0,000	0,000	Asymmetric
Hensel, 2016	Hepatitis B infection, HBsAb	221	144	473	264	0,025	0,021	Asymmetric
Barron-HbA1c diabetes, 2018	Hepatitis B infection, HBsAg	293	4	4665	13	0,014	0,015	Asymmetric
Barron-HbA1c prediabetes, 2018	Hepatitis B infection, HBsAg	293	4	4665	13	0,014	0,015	Asymmetric
Chen, 2006	Hepatitis B infection, HBsAg	29	2	727	67	0,923	1,000	Symmetric
Hensel, 2016	Hepatitis B infection, HBsAg	221	5	473	13	0,905	0,803	Symmetric
Lin, 2017	Hepatitis B infection, HBsAg	22256	635	89024	1637	0,000	0,000	Asymmetric
Swarna Nantha, 2017	Hepatitis B infection, HBsAg	404	0	359	1	0,953	0,471	Symmetric
Barron-HbA1c diabetes, 2018	Hepatitis C infection, Anti-HCV	293	4	4665	80	0,932	0,818	Symmetric
Barron-HbA1c prediabetes, 2018	Hepatitis C infection, Anti-HCV	293	4	4665	80	0,932	0,818	Symmetric
Chen, 2006	Hepatitis C infection, Anti-HCV	29	10	727	174	0,281	0,191	Symmetric
Hensel, 2016	Hepatitis C infection, Anti-HCV	221	6	473	5	0,193	0,113	Symmetric
Lin, 2017	Hepatitis C infection, Anti-HCV	22256	396	89024	835	0,000	0,000	Asymmetric
Zhussupov, 2016	Hepatitis C infection, Anti-HCV	110	4	214	4	0,553	0,451	Symmetric
Ndishimye, 2017	History of prior TB treatment	150	25	150	0	0,000	0,000	Asymmetric
Boillat-blanco, 2016	HIV infection	530	166	491	74	0,000	0,000	Asymmetric
Buskin, 1994	HIV infection	151	4	545	3	0,068	0,043	Asymmetric
Davis, 2017	HIV infection	562	7	1038	1	0,006	0,004	Asymmetric
Faurholt-Jepsen, 2011	HIV infection	803	347	350	35	0,000	0,000	Asymmetric
Faurholt-Jepsen, 2014	HIV infection	187	83	190	17	0,000	0,000	Asymmetric
Hensel, 2016	HIV infection	221	4	473	6	0,829	0,733	Symmetric
Jurcev-Savicevic, 2013	HIV infection	300	1	300	0	1,000	1,000	Symmetric
Lee, 2013	HIV infection	49903	7	49903	5	0,773	0,774	Symmetric
Leegaard, 2011	HIV infection	2950	3	14274	13	1,000	0,746	Symmetric
Lin, 2017	HIV infection	22256	5	89024	27	0,691	0,662	Symmetric
Pablos-Méndez, 1997	HIV infection	5290	912	37366	131	0,000	0,000	Asymmetric
Ting, 2014	HIV infection	296	0	722	0	NA	1,000	Symmetric
Zhussupov, 2016	HIV infection	110	2	214	0	0,219	0,115	Symmetric

Boillat-blanco, 2016	HIV treated with cART	530	51	491	6	0,000	0,000	Asymmetric
Chung, 2014	Hyperlipidaemia	10168	1714	40672	7126	0,118	0,118	Symmetric
Baker, 2012	Hypertension	1652	668	16063	1447	0,000	0,000	Asymmetric
Chung, 2014	Hypertension	10168	3931	40672	15249	0,031	0,031	Asymmetric
Leung, 2008	Hypertension	6444	4027	35672	13983	0,000	0,000	Asymmetric
Swarna Nantha, 2017	Hypertension	404	335	359	343	0,000	0,000	Asymmetric
Jurcev-Savicevic, 2013	Illicit drug use	300	3	300	1	0,616	0,624	Symmetric
Jurcev-Savicevic, 2013	Immunosuppressive therapy	300	11	300	7	0,473	0,474	Symmetric
Swarna Nantha, 2017	Ischaemic heart disease	404	9	359	17	0,088	0,071	Symmetric
Lee, 2013	Liver cirrhosis	49903	47	49903	19	0,001	0,001	Asymmetric
Lin, 2017	Liver cirrhosis	22256	697	89024	1149	0,000	0,000	Asymmetric
Shu, 2012	Liver cirrhosis	91	1	316	5	1,000	1,000	Symmetric
Ting, 2014	Liver cirrhosis	296	11	722	33	0,661	0,614	Symmetric
Wu, 2007	Liver cirrhosis	264	13	438	9	0,059	0,044	Asymmetric
Alisjahbana, 2006	Living in a crowded home	454	256	556	325	0,551	0,523	Symmetric
Baker, 2012	Living in a crowded home	1652	136	16063	1463	0,255	0,260	Symmetric
Jurcev-Savicevic, 2013	Living in a crowded home	300	139	300	94	0,000	0,000	Asymmetric
Baker, 2012	Low income	1652	520	16063	3158	0,000	0,000	Asymmetric
Baker, 2012	Lung disease	1652	173	16063	807	0,000	0,000	Asymmetric
Alisjahbana, 2006	Male gender	454	238	556	292	1,000	1,000	Symmetric
Baker, 2012	Male gender	1652	804	16063	7991	0,418	0,408	Symmetric
Barron-HbA1c diabetes, 2018	Male gender	293	164	4665	2206	0,006	0,005	Asymmetric
Barron-HbA1c prediabetes, 2018	Male gender	293	164	4665	2206	0,006	0,005	Asymmetric
Boillat-blanco, 2016	Male gender	530	341	491	254	0,000	0,000	Asymmetric
Chen, 2006	Male gender	29	21	727	422	0,178	0,177	Symmetric
Chung, 2014	Male gender	10168	6937	40672	27748	1,000	1,000	Symmetric
Corris-OGTT result or on insulin, 2012	Male gender	166	88	15	7	0,840	0,789	Symmetric
Corris, 2012	Male gender	166	88	15	7	0,840	0,789	Symmetric
Davis, 2017	Male gender	562	308	1038	431	0,000	0,000	Asymmetric
Faurholt-Jepsen, 2011	Male gender	803	497	350	193	0,037	0,036	Asymmetric
Faurholt-Jepsen, 2014	Male gender	187	115	190	110	0,543	0,529	Symmetric
Haraldsdottir, 2015	Male gender	110	61	572	213	0,001	0,000	Asymmetric
Hensel, 2016	Male gender	221	127	473	254	0,397	0,369	Symmetric
Kubiak, 2019	Male gender	919	729	1113	329	0,000	0,000	Asymmetric
Lai, 2014	Male gender	11366	7535	45464	30140	1,000	1,000	Symmetric
Lee, 2013	Male gender	49903	26202	49903	26202	1,000	1,000	Symmetric
Lee, 2016	Male gender	11260	4742	110782	38801	0,000	0,000	Asymmetric
Leegaard, 2011	Male gender	2950	1567	14274	7610	0,863	0,855	Symmetric
Leung, 2008	Male gender	6444	2223	35672	12378	0,764	0,765	Symmetric
Lin, 2017	Male gender	22256	12211	89024	48844	1,000	1,000	Symmetric
Lin, 2019	Male gender	2948	1468	453	109	0,000	0,000	Asymmetric
Mori, 1992	Male gender	46	30	46	21	0,093	0,093	Symmetric

Ndishimye, 2017	Male gender	150	118	150	118	1,000	1,000	Symmetric
Pablos-Méndez, 1997	Male gender	5290	3522	37366	21318	0,000	0,000	Asymmetric
Pealing, 2015	Male gender	222731	122594	1218616	647287	0,000	0,000	Asymmetric
Pereira, 2016	Male gender	323	197	323	196	1,000	1,000	Symmetric
Pérez, 2006	Male gender	4915	2672	70808	34610	0,000	0,000	Asymmetric
Shen, 2014	Male gender	5195	2478	20780	9912	1,000	1,000	Symmetric
Shimouchi, 2020	Male gender	192	179	190	182	0,383	0,370	Symmetric
Shu, 2012	Male gender	91	55	316	162	0,154	0,152	Symmetric
Swarna Nantha, 2017	Male gender	404	169	359	206	0,000	0,000	Asymmetric
Ting, 2014	Male gender	296	174	722	360	0,012	0,011	Asymmetric
Viney, 2015	Male gender	275	144	499	235	0,184	0,176	Symmetric
Wang, 2013	Male gender	6382	4631	6675	3712	0,000	0,000	Asymmetric
Wardhani, 2019	Male gender	2259	874	31993	15534	0,000	0,000	Asymmetric
Wu, 2007	Male gender	264	200	438	234	0,000	0,000	Asymmetric
Zhussupov, 2016	Male gender	110	62	214	87	0,010	0,009	Asymmetric
Buskin, 1994	Malignant disease	151	6	545	6	0,041	0,027	Asymmetric
Chen, 2006	Malignant disease	29	4	727	55	0,383	0,274	Symmetric
Golub, 2019	Malignant disease	52820	90	766231	1073	0,083	0,083	Symmetric
Jurcev-Savicevic, 2013	Malignant disease	300	15	300	5	0,041	0,038	Asymmetric
Lee, 2013	Malignant disease	49903	1015	49903	922	0,035	0,035	Asymmetric
Lee, 2014	Malignant disease	300	36	300	24	0,134	0,134	Symmetric
Lee, 2016	Malignant disease	11260	319	110782	1789	0,000	0,000	Asymmetric
Leung, 2008	Malignant disease	6444	38	35672	249	0,373	0,366	Symmetric
Lin, 2017	Malignant disease	22256	625	89024	2118	0,000	0,000	Asymmetric
Pérez, 2006	Malignant disease	4915	357	70808	6203	0,000	0,000	Asymmetric
Shu, 2012	Malignant disease	91	10	316	19	0,163	0,110	Symmetric
Ting, 2014	Malignant disease	296	106	722	245	0,617	0,611	Symmetric
Wu, 2007	Malignant disease	264	6	438	9	1,000	1,000	Symmetric
Baker, 2012	Marital status, Single	1652	1241	16063	8889	0,000	0,000	Asymmetric
Davis, 2017	Marital status, Single	562	152	1038	144	0,000	0,000	Asymmetric
Faurholt-Jepsen, 2011	Marital status, Single	803	218	350	89	0,597	0,563	Symmetric
Jurcev-Savicevic, 2013	Marital status, Single	300	95	300	126	0,011	0,011	Asymmetric
Kubiak, 2019	Marital status, Single	919	246	1113	411	0,000	0,000	Asymmetric
Lee, 2016	Marital status, Single	11260	311	110782	5752	0,000	0,000	Asymmetric
Leegaard, 2011	Marital status, Single	2950	660	14274	2705	0,000	0,000	Asymmetric
Leung, 2008	Marital status, Single	6444	193	35672	1427	0,000	0,000	Asymmetric
Ndishimye, 2017	Marital status, Single	150	67	150	62	0,641	0,641	Symmetric
Wang, 2013	Marital status, Single	6382	1221	6675	754	0,000	0,000	Asymmetric
Pérez, 2006	Nutrition deficit	4915	464	70808	1051	0,000	0,000	Asymmetric
Ting, 2014	Organ transplantation	296	0	722	12	0,056	0,023	Asymmetric
Swarna Nantha, 2017	Osteoarthritis	404	1	359	1	1,000	1,000	Symmetric
Jurcev-Savicevic, 2013	Other chronic diseases	300	130	300	133	0,869	0,869	Symmetric

Buskin, 1994	Pancreatitis	151	2	545	3	0,651	0,298	Symmetric
Buskin, 1994	Partial gastrectomy	151	4	545	3	0,068	0,043	Asymmetric
Ndishimye, 2017	Persistent cough	150	116	150	49	0,000	0,000	Asymmetric
Hossain, 2014	Physical activity	50	15	50	10	0,356	0,356	Symmetric
Lai, 2014	Pneumoconiosis	11366	198	45464	305	0,000	0,000	Asymmetric
Lee, 2013	Pneumoconiosis	4993	3	49903	1	0,000	0,003	Asymmetric
Lee, 2016	Pneumoconiosis	11260	103	110782	641	0,000	0,000	Asymmetric
Lin, 2017	Pneumoconiosis	22256	183	89024	637	0,105	0,096	Symmetric
Wu, 2007	Pneumoconiosis	264	44	438	28	0,000	0,000	Asymmetric
Lee, 2014	Poly-drug resistant	300	9	300	4	0,262	0,262	Symmetric
Ting, 2014	Post gastrectomy	296	10	722	9	0,043	0,037	Asymmetric
Ndishimye, 2017	Presence of TB patient in household	150	16	150	4	0,011	0,009	Asymmetric
Jurcev-Savicevic, 2013	Previous hospitalizations	300	188	300	190	0,933	0,933	Symmetric
Leung, 2008	Previous hospitalizations	6444	1095	35672	4744	0,000	0,000	Asymmetric
Boillat-blanco, 2016	Previously known HIV	530	86	491	10	0,000	0,000	Asymmetric
Davis, 2017	Prisoners	562	21	1038	15	0,006	0,004	Asymmetric
Jurcev-Savicevic, 2013	Prisoners	300	13	300	20	0,283	0,283	Symmetric
Baker, 2012	Receiving government subsidy	1652	474	16063	3006	0,000	0,000	Asymmetric
Baker, 2012	Residence in an indigenous community	1652	127	16063	1176	0,621	0,586	Symmetric
Viney, 2015	Self-reported comorbidities	275	14	499	73	0,000	0,000	Asymmetric
Kubiak, 2019	Self-reported history of renal failure	919	1	1113	3	0,756	0,631	Symmetric
Barron-HbA1c diabetes, 2018	Self-reported Hypertension	293	114	4665	1448	0,006	0,006	Asymmetric
Barron-HbA1c prediabetes, 2018	Self-reported Hypertension	293	114	4665	1448	0,006	0,006	Asymmetric
Baker, 2012	Smoke Exposure	1652	336	16063	3557	0,098	0,092	Symmetric
Barron-HbA1c diabetes, 2018	Smoke Exposure	293	65	4665	902	0,278	0,254	Symmetric
Barron-HbA1c prediabetes, 2018	Smoke Exposure	293	65	4665	902	0,278	0,254	Symmetric
Boillat-blanco, 2016	Smoke Exposure	530	149	491	99	0,004	0,003	Asymmetric
Buskin, 1994	Smoke Exposure	151	69	545	209	0,124	0,111	Symmetric
Corris-OGTT result or on insulin, 2012	Smoke Exposure	166	62	15191	5742	0,969	0,936	Symmetric
Corris, 2012	Smoke Exposure	166	62	15191	5742	0,969	0,936	Symmetric
Davis, 2017	Smoke Exposure	562	138	1038	93	0,000	0,000	Asymmetric
Golub, 2019	Smoke Exposure	52820	27366	766231	411645	0,000	0,000	Asymmetric
Haraldsdottir, 2015	Smoke Exposure	110	9	572	30	0,322	0,259	Symmetric
Hensel, 2016	Smoke Exposure	221	50	473	107	1,000	1,000	Symmetric
Jurcev-Savicevic, 2013	Smoke Exposure	300	107	300	68	0,001	0,001	Asymmetric
Jurcev-Savicevic, 2013	Smoke Exposure	300	68	300	107	0,001	0,001	Asymmetric
Kubiak, 2019	Smoke Exposure	919	215	1113	107	0,000	0,000	Asymmetric
Lee, 2014	Smoke Exposure	300	146	300	116	0,017	0,017	Asymmetric
Lee, 2016	Smoke Exposure	11260	1821	110782	16280	0,000	0,000	Asymmetric
Leung, 2008	Smoke Exposure	6444	490	35672	3317	0,000	0,000	Asymmetric
Lin, 2019	Smoke Exposure	2948	433	453	31	0,000	0,000	Asymmetric
Ndishimye, 2017	Smoke Exposure	150	97	150	45	0,000	0,000	Asymmetric

Pealing, 2015	Smoke Exposure	222731	42414	1218616	244247	0,000	0,000	Asymmetric
Shimouchi, 2020	Smoke Exposure	192	108	190	120	0,203	0,177	Symmetric
Shu, 2012	Smoke Exposure	91	8	316	16	0,281	0,206	Symmetric
Ting, 2014	Smoke Exposure	296	121	722	195	0,000	0,000	Asymmetric
Wang, 2013	Smoke Exposure	6382	900	6675	379	0,000	0,000	Asymmetric
Zhussupov, 2016	Smoke Exposure	110	41	214	59	0,096	0,077	Symmetric
Lee, 2016	Steroïde use	11260	387	110782	2427	0,000	0,000	Asymmetric
Hensel, 2016	Syphilis	221	3	473	10	0,701	0,765	Symmetric
Swarna Nantha, 2017	Thyroid disorder	404	4	359	8	0,280	0,244	Symmetric
Barron-HbA1c diabetes, 2018	Total Bilirubin (mg/dL), Not Normal	293	2	4665	62	0,599	0,588	Symmetric
Barron-HbA1c prediabetes, 2018	Total Bilirubin (mg/dL), Not Normal	293	2	4665	62	0,599	0,588	Symmetric
Jurcev-Savicevic, 2013	Transplantation	300	1	300	1	1,000	1,000	Symmetric
Lee, 2013	Transplantation	49903	22	49903	8	0,018	0,016	Asymmetric
Baker, 2012	Unemployed	1652	1090	16063	7369	0,000	0,000	Asymmetric
Jurcev-Savicevic, 2013	Unemployed	300	22	300	70	0,000	0,000	Asymmetric
Ndishimye, 2017	Unemployed	150	121	150	100	0,009	0,009	Asymmetric
Davis, 2017	Used marijuana in the past 90 days	562	8	1038	4	0,046	0,031	Asymmetric
Leung, 2008	Weight loss	6444	251	35672	892	0,000	0,000	Asymmetric
Ndishimye, 2017	Weight loss	150	114	150	92	0,009	0,009	Asymmetric
