# Supplementary material for: Diabetes mellitus and tuberculosis, a systematic review and meta-analysis with sensitivity analysis for studies comparable for confounders
Source: PLoS One. 2021 Dec 10;16(12):e0261246. doi: 10.1371/journal.pone.0261246 (PMC8664214; doi:10.1371/journal.pone.0261246)
Supplement: S7 Table — (PDF) [file pone.0261246.s010.pdf]

S7 Table. P-value of Student test for quantitative confounding factors

Author, Year	Quantitative confounding factors	Total number of reference group	Mean for reference group	SD for reference group	Total number of control group	Mean for control group	SD for control group	P-Value for Student Test with unequal variance	Status
Baker, 2012	Age	1652	59,8	13,3	16063	36,6	18,7	0,000	Asymmetric
Boillat-blanco, 2016	Age	530	35,9	12,0	491	36,7	13,0	0,154	Symmetric
Chen, 2006	Age	29	42,5	15,2	722	40,6	12,1	0,256	Symmetric
Davis, 2017	Age	562	35,4	13,1	1038	40,8	13,4	0,000	Asymmetric
Faurholt-Jepsen, 2011	Age	803	34,8	11,9	350	33,8	12,0	0,096	Symmetric
Faurholt-Jepsen, 2014	Age	187	34,4	12,7	190	34,0	11,9	0,376	Symmetric
Hensel, 2016	Age	221	36,1	14,2	473	33,1	10,9	0,003	Asymmetric
Kubiak, 2019	Age	919	44,9	14,0	1113	36,8	14,4	0,000	Asymmetric
Lee, 2013	Age	49903	61,9	14,2	49903	61,9	14,2	0,500	Symmetric
Leegaard, 2011	Age	2950	53,4	26,2	14274	53,6	26,3	0,307	Symmetric
Lin, 2017	Age	22256	56,8	13,4	89024	56,8	13,7	0,500	Symmetric
Lin, 2019	Age	2948	61,5	9,3	453	51,3	10,5	0,000	Asymmetric
Ndishimye, 2017	Age	150	43,9	7,8	150	42,5	9,3	0,079	Symmetric
Pealing, 2015	Age	222731	62,6	14,8	1218616	63,3	14,5	0,000	Asymmetric
Pereira, 2016	Age	323	38,5	14,2	323	38,5	14,3	0,500	Symmetric
Shen, 2014	Age	5195	18,9	10,2	20780	23,8	9,8	0,000	Asymmetric
Shu, 2012	Age	91	64,9	11,0	316	60,0	13,5	0,000	Asymmetric
Swarna Nantha, 2017	Age	404	61,7	9,8	359	63,0	10,0	0,035	Asymmetric
Wang, 2013	Age	6382	50,4	18,6	6675	57,8	16,4	0,000	Asymmetric
Wu, 2007	Age	264	61,0	19,1	438	55,5	18,5	0,000	Asymmetric
Faurholt-Jepsen, 2014	Alpha-1-acid glycoprotein, g/l	187	2,6	0,8	190	0,7	0,3	0,000	Asymmetric
Alisjahbana, 2006	Body mass index	454	17,3	4,9	556	24,1	4,4	0,000	Asymmetric
Baker, 2012	Body mass index	1652	25,0	3,5	16063	22,4	3,6	0,000	Asymmetric
Boillat-blanco, 2016	Body mass index	530	19,6	4,0	491	25,4	5,0	0,000	Asymmetric
Faurholt-Jepsen, 2011	Body mass index	803	18,4	2,7	350	22,6	4,1	0,000	Asymmetric
Faurholt-Jepsen, 2014	Body mass index	187	18,3	2,4	190	22,4	3,8	0,000	Asymmetric
Hensel, 2016	Body mass index	221	24,0	4,5	473	24,0	4,5	0,427	Symmetric
Wang, 2013	Body mass index	6382	20,9	2,8	6675	22,5	3,0	0,000	Asymmetric
Faurholt-Jepsen, 2014	CD4 count, cells/ ìl	187	483,8	340,4	190	577,5	318,2	0,003	Asymmetric
Davis, 2017	Cigarettes smoked in a week	562	14,0	38,9	1038	12,2	38,0	0,192	Symmetric

Chen, 2006	Dialysis duration	29	38,1	42,6	722	27,2	52,8	0,095	Symmetric
Shu, 2012	Dialysis duration	91	4,3	3,6	316	4,7	4,3	0,187	Symmetric
Wang, 2013	Diastolic blood pressure	6382	76,7	7,7	6675	79,3	9,8	0,000	Asymmetric
Boillat-blanco, 2016	Hemoglobin	530	10,8	2,2	491	12,9	1,7	0,000	Asymmetric
Wang, 2013	Hemoglobin	6382	131,9	17,3	6675	131,1	43,5	0,077	Symmetric
Wang, 2013	High Density Lipoprotein Cholesterol	6382	1,5	0,6	6675	1,6	0,8	0,000	Asymmetric
Faurholt-Jepsen, 2014	Lymphocytes, cells/ ìl	187	1,8	0,8	190	2,1	0,6	0,000	Asymmetric
Faurholt-Jepsen, 2014	Neutrophil granulocytes, cells/ ìl	187	4,5	1,8	190	2,1	1,0	0,000	Asymmetric
Shu, 2012	Serum albumin, g/dL	91	4,1	0,3	316	4,0	0,4	0,005	Asymmetric
Wang, 2013	Systolic blood pressure	6382	119,7	11,4	6675	124,0	15,0	0,000	Asymmetric
Wang, 2013	Total cholesterol	6382	4,4	1,1	6675	4,7	1,2	0,000	Asymmetric
Wang, 2013	Triglyceride	6382	1,2	1,1	6675	1,3	0,9	0,000	Asymmetric
Kubiak, 2019	Years of schooling	919	6,9	4,7	1113	8,0	5,1	0,000	Asymmetric
