# Supplementary material for: Diabetes mellitus and tuberculosis, a systematic review and meta-analysis with sensitivity analysis for studies comparable for confounders
Source: PLoS One. 2021 Dec 10;16(12):e0261246. doi: 10.1371/journal.pone.0261246 (PMC8664214; doi:10.1371/journal.pone.0261246)
Supplement: S8 Table — (PDF) [file pone.0261246.s011.pdf]

S8 Table. Subgroup analyses of the association between diabetes and tuberculosis.

	OR (95%CI)	95% Prediction interval	N Studies	N HCC cases	N controls	H (95%CI)	I^2 (95%CI)	P-value heterogeneity	P-value Egger test	P-value subgroup difference
**Cohort studies**										
**Country**										< 0.001
China	1.8 [1.5-2.3]	[0.8-4.2]	7	147051	460838	2.6 [1.9-3.6]	85.4 [71.8-92.4]	< 0.001	0,043	
Korean	3.5 [3-4.1]	NA	1	8015	806698	NA	NA	1	NA	
United Kingdom	1.3 [1.1-1.6]	NA	1	222731	1218616	NA	NA	1	NA	
United States of America	1.8 [1.7-1.9]	NA	1	52820	766231	NA	NA	1	NA	
**UNSD Region**										0,002
Eastern Asia	2 [1.6-2.7]	[0.8-5.3]	8	155066	1267536	4.5 [3.6-5.7]	95.1 [92.4-96.9]	< 0.001	0,073	
Northern America	1.8 [1.7-1.9]	NA	1	52820	766231	NA	NA	1	NA	
Northern Europe	1.3 [1.1-1.6]	NA	1	222731	1218616	NA	NA	1	NA	
**Country income level**										0,029
High-income economies	2.1 [1.6-2.7]	[0.8-5.4]	8	359316	3009135	4.3 [3.5-5.5]	94.7 [91.6-96.6]	< 0.001	0,491	
Upper-middle-income economies	1.5 [1.4-1.6]	NA	2	71301	243248	1.6 [1-3.3]	61.5 [0-91.1]	0,107	NA	
**TB stage**										0,162
Active tuberculosis	1.9 [1.5-2.4]	[0.8-4.5]	9	427669	3251930	4.4 [3.5-5.4]	94.8 [92-96.6]	< 0.001	0,452	
Latent tuberculosis infection	2.5 [1.8-3.4]	NA	1	2948	453	NA	NA	1	NA	
**Controls**										0,014
Non-DM	2 [1.6-2.5]	[0.8-4.8]	9	381572	3098159	4.1 [3.3-5.2]	94.2 [91-96.2]	< 0.001	0,471	
Presumed healthy controls	1.4 [1.3-1.6]	NA	1	49045	154224	NA	NA	1	NA	
**Case control studies**										
**Country**										< 0.001
Bangladesh	3 [0.7-12]	NA	1	50	50	NA	NA	1	NA	
Brazil	2.7 [1.5-4.7]	NA	1	323	323	NA	NA	1	NA	

	OR (95%CI)	95% Prediction interval	N Studies	N HCC cases	N controls	H (95%CI)	I² (95%CI)	P-value heterogeneity	P-value Egger test	P-value subgroup difference
China	1.6 [1.5-1.6]	[1.2-2]	3	21834	86436	1 [1-2]	0 [0-76.2]	0,646	0,775	
Croatia	1.9 [1-3.5]	NA	1	300	300	NA	NA	1	NA	
Denmark	1.4 [1.2-1.7]	NA	1	2950	14247	NA	NA	1	NA	
Guinea-Bissau	1.4 [0.4-5.2]	NA	1	110	572	NA	NA	1	NA	
India	4.4 [1.4-13.3]	NA	1	46	46	NA	NA	1	NA	
Indonesia	4.6 [2.6-7.8]	NA	1	454	556	NA	NA	1	NA	
Japan	1.2 [0.7-1.9]	NA	1	192	190	NA	NA	1	NA	
Kazakhstan	9.3 [4.8-17.9]	NA	2	672	1252	1 NA	0 NA	0,732	NA	
Kiribati – Republic of Kiribati	2.5 [1.8-3.5]	NA	1	275	499	NA	NA	1	NA	
Romania	3.3 [1.4-8.1]	NA	1	150	150	NA	NA	1	NA	
Tanzania	3 [1.7-5.3]	NA	2	1333	841	3.1 [1.6-5.9]	89.4 [60.5-97.2]	0,002	NA	
United Kingdom	2.7 [1.7-4.1]	NA	1	497	1966	NA	NA	1	NA	
United States of America	2.4 [1.8-3.1]	[0.9-6.4]	5	10908	162510	3.3 [2.3-4.7]	90.9 [81.7-95.5]	< 0.001	0,826	
**UNSD Region**										< 0.001
Central Asia	9.3 [4.8-17.9]	NA	2	672	1252	1 NA	0 NA	0,732	NA	
Eastern Africa	3 [1.7-5.3]	NA	2	1333	841	3.1 [1.6-5.9]	89.4 [60.5-97.2]	0,002	NA	
Eastern Asia	1.6 [1.5-1.6]	[1.5-1.7]	4	22026	86626	1 [1-2.3]	0 [0-80.7]	0,498	0,502	
Eastern Europe	3.3 [1.4-8.1]	NA	1	150	150	NA	NA	1	NA	
Northern America	2.4 [1.8-3.1]	[0.9-6.4]	5	10908	162510	3.3 [2.3-4.7]	90.9 [81.7-95.5]	< 0.001	0,826	
Northern Europe	1.8 [1.2-2.8]	NA	2	3447	16213	2.6 [1.3-5.3]	85.6 [41.9-96.4]	0,008	NA	
Oceania	2.5 [1.8-3.5]	NA	1	275	499	NA	NA	1	NA	
South America	2.7 [1.5-4.7]	NA	1	323	323	NA	NA	1	NA	
Southeastern Asia	4.6 [2.6-7.8]	NA	1	454	556	NA	NA	1	NA	
Southern Asia	3.8 [1.6-9]	NA	2	96	96	1 NA	0 NA	0,674	NA	
Southern Europe	1.9 [1-3.5]	NA	1	300	300	NA	NA	1	NA	
West Africa	1.4 [0.4-5.2]	NA	1	110	572	NA	NA	1	NA	

	OR (95%CI)	95% Prediction interval	N Studies	N HCC cases	N controls	H (95%CI)	I² (95%CI)	P-value heterogeneity	P-value Egger test	P-value subgroup difference
**Country income level**										0,049
High-income economies	1.9 [1.6-2.4]	[1-3.9]	11	25299	205144	4.6 [3.8-5.5]	95.3 [93.1-96.7]	< 0.001	0,683	
Low-income economies	1.4 [0.4-5.2]	NA	1	110	572	NA	NA	1	NA	
Lower-middle-income economies	2.9 [2.1-4.1]	[1-8.2]	5	1704	1436	1.7 [1-2.7]	63.5 [3.6-86.2]	0,027	0,973	
Upper-middle-income economies	3.6 [2.1-5.9]	[0.7-18.7]	6	12981	62786	3.1 [2.3-4.3]	89.7 [80.4-94.6]	< 0.001	0,006	
**TB stage**										0,81
Active tuberculosis	2.4 [1.9-2.9]	[1-5.7]	22	39819	269439	3.9 [3.3-4.4]	93.3 [91.1-94.9]	< 0.001	0,108	
Latent tuberculosis infection	2.5 [1.8-3.5]	NA	1	275	499	NA	NA	1	NA	
**Controls**										0,208
Non-TB diseases	2.1 [1.8-2.6]	[1.1-4]	14	26474	185479	4 [3.3-4.7]	93.6 [90.9-95.5]	< 0.001	0,428	
Presumed healthy controls	2.9 [1.9-4.4]	[0.7-12]	9	13620	84459	3.2 [2.5-4.1]	90.1 [83.5-94.1]	< 0.001	0,024	
**Cross sectional studies**										
**Country**										< 0.001
China	2.2 [1.4-3.5]	[0.5-10]	6	8714	24941	3.5 [2.6-4.8]	91.9 [85.2-95.6]	< 0.001	0,078	
India	7.2 [5.4-9.5]	NA	1	919	1113	NA	NA	1	NA	
Indonesia	3.9 [2.5-6]	NA	1	2260	31998	NA	NA	1	NA	
Malaysia	1 [0.7-1.3]	NA	1	404	359	NA	NA	1	NA	
Tanzania	1.9 [1.1-3.2]	NA	1	187	190	NA	NA	1	NA	
Thailand	1.6 [0.3-9.2]	NA	1	36	114	NA	NA	1	NA	
United States of America	2.8 [1.6-4.7]	[0.3-22.3]	5	20529	15809	5.2 [4-6.8]	96.3 [93.7-97.8]	< 0.001	0,104	
**UNSD Region**										< 0.001
Eastern Africa	1.9 [1.1-3.2]	NA	1	187	190	NA	NA	1	NA	
Eastern Asia	2.2 [1.4-3.5]	[0.5-10]	6	8714	24941	3.5 [2.6-4.8]	91.9 [85.2-95.6]	< 0.001	0,078	
Northern America	2.8 [1.6-4.7]	[0.3-22.3]	5	20529	15809	5.2 [4-6.8]	96.3 [93.7-97.8]	< 0.001	0,104	
Southeastern Asia	1.8 [0.8-4.2]	[0-22704.1]	3	2700	32471	3.6 [2.2-5.6]	92.1 [80-96.9]	< 0.001	0,607	
Southern Asia	7.2 [5.4-9.5]	NA	1	919	1113	NA	NA	1	NA	

	OR (95%CI)	95% Prediction interval	N Studies	N HCC cases	N controls	H (95%CI)	I² (95%CI)	P-value heterogeneity	P-value Egger test	P-value subgroup difference
**Country income level**										0,28
High-income economies	2.9 [1.9-4.4]	[0.7-12.4]	7	22210	32599	4.3 [3.3-5.5]	94.6 [91.1-96.7]	< 0.001	0,107	
Lower-middle-income economies	3.9 [1.5-9.6]	NA	2	1106	1303	4.3 [2.5-7.6]	94.7 [83.8-98.3]	< 0.001	NA	
Upper-middle-income economies	1.9 [1.2-3]	[0.4-9.2]	7	9733	40622	3.6 [2.7-4.7]	92.3 [86.6-95.5]	< 0.001	0,156	
**TB stage**										0,738
Active tuberculosis	2.6 [1.7-4.1]	[0.6-11.9]	8	29549	61575	5 [4-6.1]	95.9 [93.8-97.3]	< 0.001	0,109	
Latent tuberculosis infection	2.4 [1.5-3.8]	[0.4-12.9]	8	3500	12949	4 [3.1-5]	93.6 [89.7-96.1]	< 0.001	0,833	
**Controls**										0,382
Non-DM	1.9 [1.1-3.3]	[0.1-25.8]	4	20703	25164	4.7 [3.4-6.5]	95.5 [91.4-97.7]	< 0.001	0,52	
Non-TB diseases	2.6 [1.6-4]	[0.5-12.8]	8	10940	47505	4.6 [3.7-5.7]	95.3 [92.7-97]	< 0.001	0,053	
Presumed healthy controls	3.4 [1.9-6.2]	[0.3-43.3]	4	1406	1855	2.8 [1.8-4.4]	87.6 [70.6-94.8]	< 0.001	0,254	
